# Supplementary material for: Encoded metabolic remodeling amplifies drug resistance in Mycobacterium tuberculosis
Source: PLoS Pathog. 2026 Aug 14;22(8):e1014237. doi: 10.1371/journal.ppat.1014237 (PMC13497261; doi:10.1371/journal.ppat.1014237)
Supplement: Supplementary file 1 [file ppat.1014237.s001.pdf]

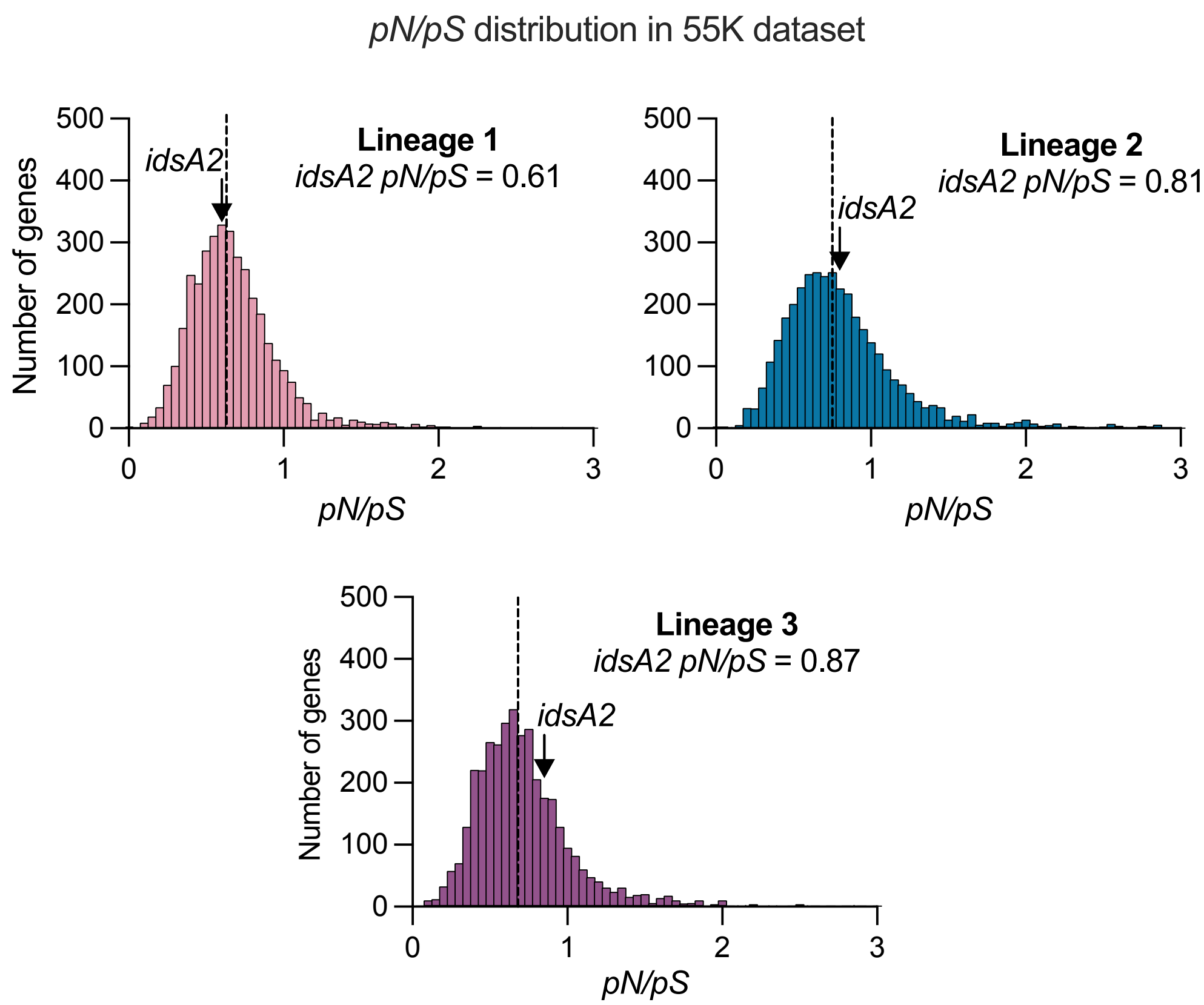

S1 Fig: Distribution of *pN/pS* ratios for L1, L2, and L3. Histograms of *pN/pS* ratios for all genes in Mtb from the Lineages 1, 2, and 3 from the 55K dataset. Median values indicated by dotted line are 0.64, 0.75, and 0.67 respectively. *idsA2 pN/pS* values indicated are 0.61, 0.81 and 0.87 respectively.
