# Supplementary material for: Encoded metabolic remodeling amplifies drug resistance in Mycobacterium tuberculosis
Source: PLoS Pathog. 2026 Aug 14;22(8):e1014237. doi: 10.1371/journal.ppat.1014237 (PMC13497261; doi:10.1371/journal.ppat.1014237)
Supplement: Supplementary file 2 [file ppat.1014237.s002.pdf]

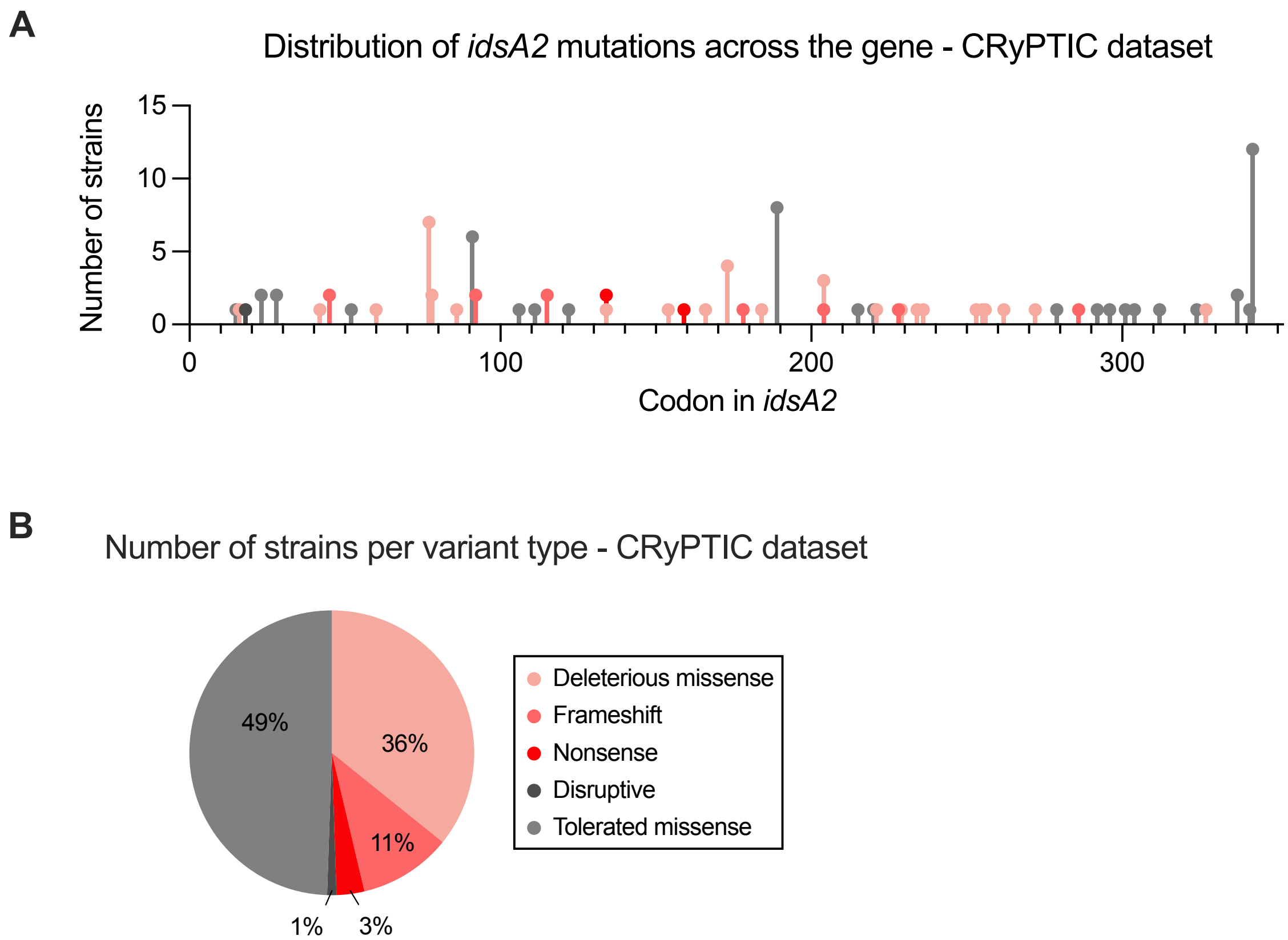

S2 Fig: *IdsA2* variants in the CRyPTIC dataset. (A) The number of strains per codon from Lineage 4 of the CRyPTIC dataset with disruptive in-frame deletions or insertions, frameshift, deleterious or tolerated missense, or nonsense mutations in *idsA2*. (B) Pie chart of total percentages of each variant type among Lineage 4 strains in the CRyPTIC dataset.
