# Supplementary material for: Encoded metabolic remodeling amplifies drug resistance in Mycobacterium tuberculosis
Source: PLoS Pathog. 2026 Aug 14;22(8):e1014237. doi: 10.1371/journal.ppat.1014237 (PMC13497261; doi:10.1371/journal.ppat.1014237)
Supplement: Supplementary file 5 [file ppat.1014237.s005.pdf]

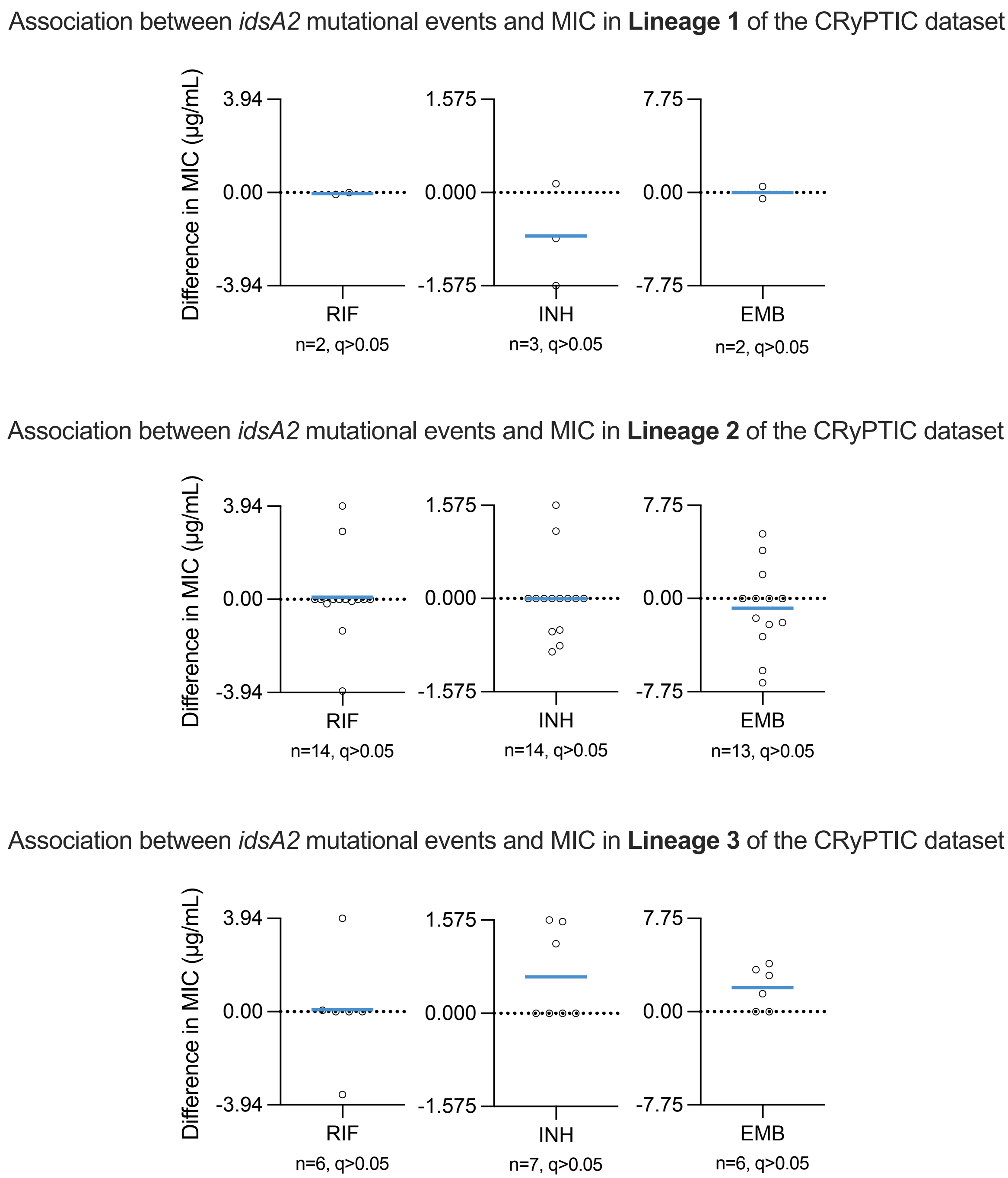

S5 Fig: Association between *idsA2* mutational event and MIC change in Lineages 1, 2, and 3 of the CRyPTIC dataset. Difference in MIC of rifampicin (RIF), isoniazid (INH), and ethambutol (EMB) associated with *idsA2* mutational events in Lineage 1, 2, and 3 data from the CRyPTIC consortium. Each dot represents the MIC difference attributed to an *idsA2* mutational event relative to its nearest neighbor strains. The blue line indicates the average MIC difference across all *idsA2* mutational events. A permutation test was performed to assess the significance of the average difference associated with *idsA2,* and a multiple test correction was performed with an FDR of 1% across each lineage.
