# Supplementary material for: Encoded metabolic remodeling amplifies drug resistance in Mycobacterium tuberculosis
Source: PLoS Pathog. 2026 Aug 14;22(8):e1014237. doi: 10.1371/journal.ppat.1014237 (PMC13497261; doi:10.1371/journal.ppat.1014237)
Supplement: Supplementary file 6 [file ppat.1014237.s006.pdf]

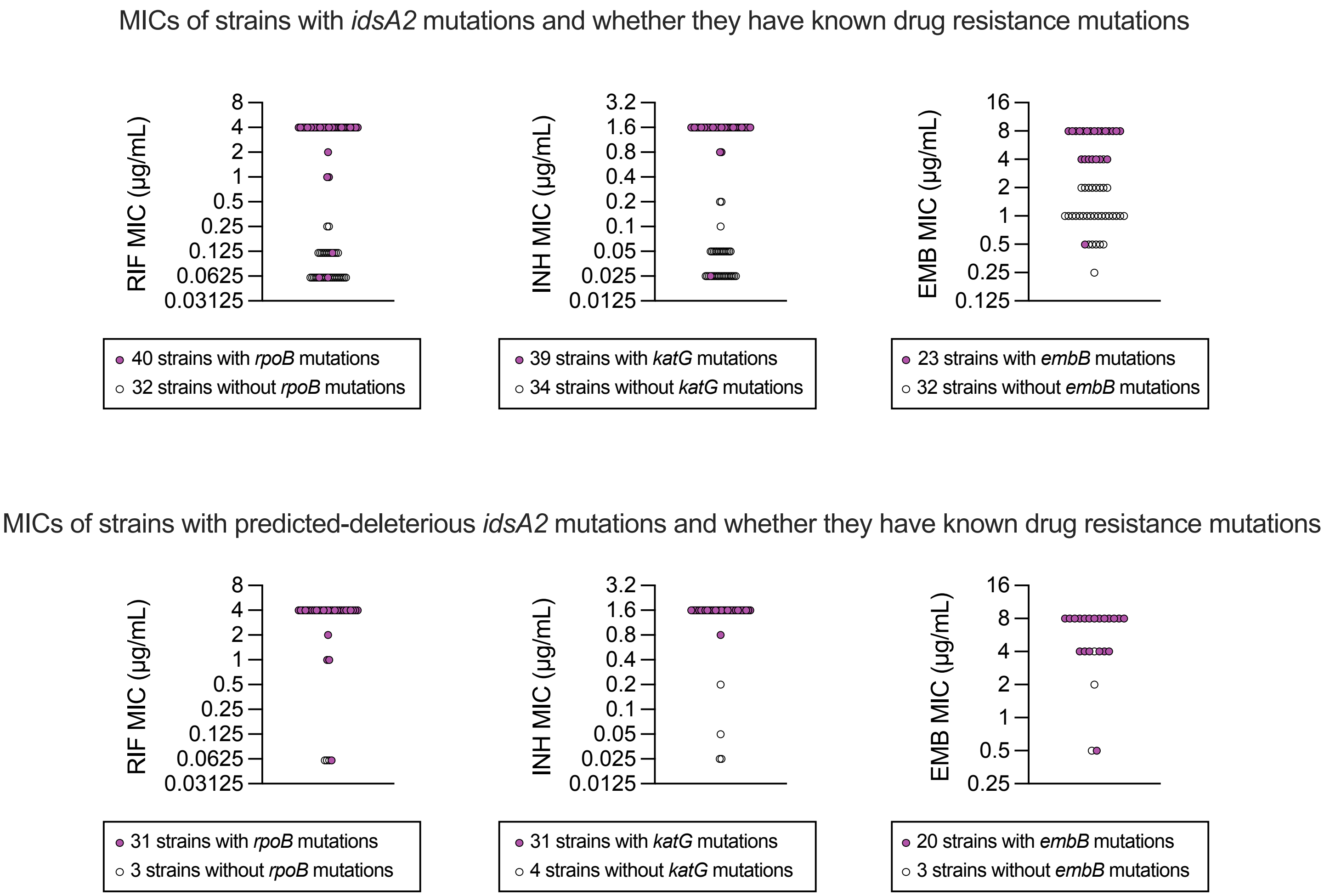

S6 Fig: MIC distribution and presence of drug resistance mutations in *idsA2* mutant strains from the CRyPTIC dataset. Rifampicin (RIF), isoniazid (INH), and ethambutol (EMB) MICs for *idsA2* mutant strains in Lineage 4 data from the CRyPTIC consortium. Each dot is an *idsA2* mutant strain. The dot is pink if there is a mutation in the indicated gene associated with drug resistance for the plotted drug (*rpoB* for RIF, *katG* for INH, and *embB* for EMB) in that strain.
