# Supplementary material for: Encoded metabolic remodeling amplifies drug resistance in Mycobacterium tuberculosis
Source: PLoS Pathog. 2026 Aug 14;22(8):e1014237. doi: 10.1371/journal.ppat.1014237 (PMC13497261; doi:10.1371/journal.ppat.1014237)
Supplement: Supplementary file 7 [file ppat.1014237.s007.pdf]

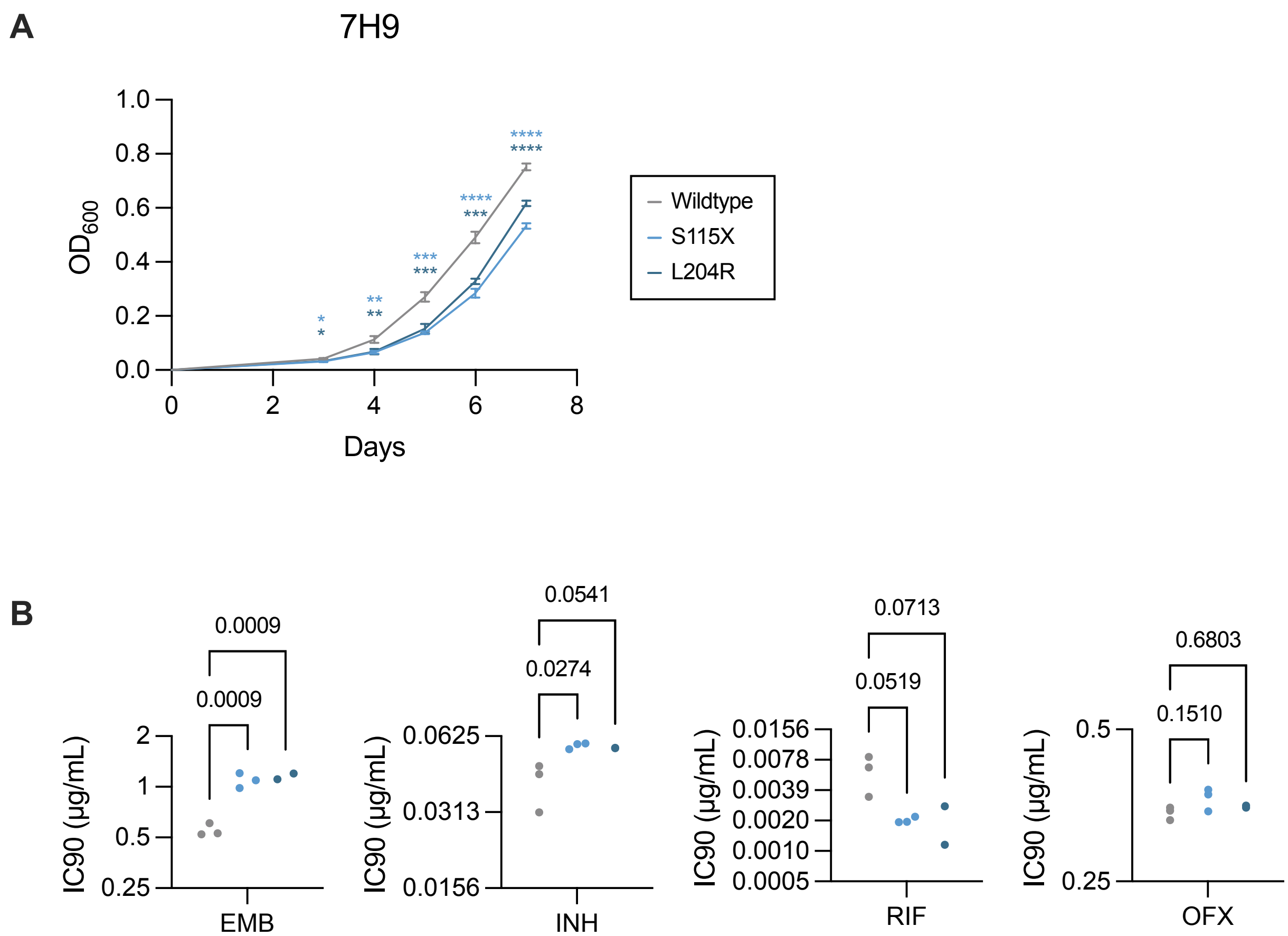

S7 Fig: Growth curve and IC90 statistics. (A) Growth curves of wildtype and *idsA2* mutant strains in standard 7H9 media. Differences between each mutant and wildtype were tested by performing ordinary one-way ANOVA with Dunnett's multiple comparisons test for each day. * $p<0.05$, ** $p<0.01$, *** $p<0.001$ **** $p<0.0001$. (B) Plots of IC90 values for each strain separated by drug: ethambutol (EMB), isoniazid (INH), rifampicin (RIF), and ofloxacin (OFX). Differences between each mutant and wildtype were tested by performing ordinary one-way ANOVA with Dunnett's multiple comparisons test.
