# Supplementary material for: Encoded metabolic remodeling amplifies drug resistance in Mycobacterium tuberculosis
Source: PLoS Pathog. 2026 Aug 14;22(8):e1014237. doi: 10.1371/journal.ppat.1014237 (PMC13497261; doi:10.1371/journal.ppat.1014237)
Supplement: Supplementary file 8 [file ppat.1014237.s008.pdf]

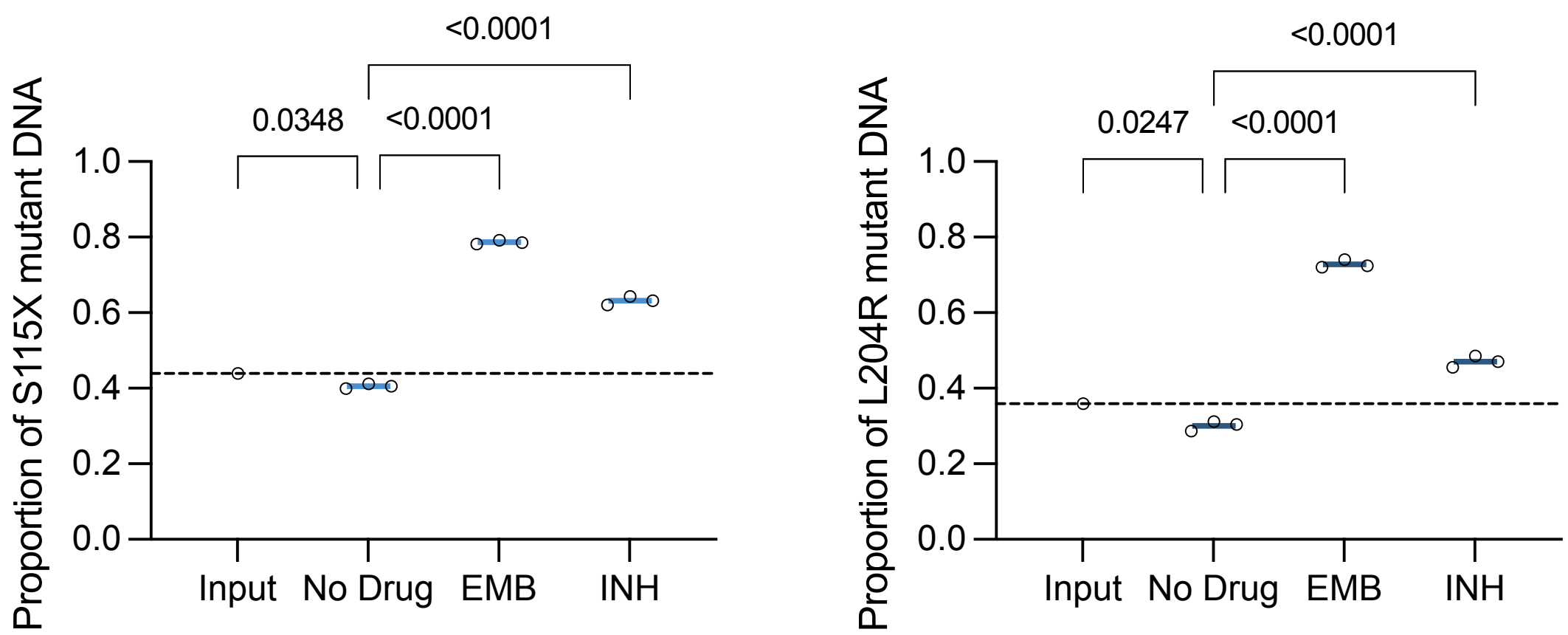

S8 Fig: Competition assay. The proportion of mutant DNA in a wildtype to single mutant competition assay either at initial pooling (input), or after 5 days of growth in 7H9 without drug, 0.55 µg/mL ethambutol, or 0.04 µg/mL isoniazid. S115X is shown to the left, L204R is shown to the right. Dotted line at input. Differences between the no drug condition and other conditions were tested by performing ordinary one-way ANOVA with Šídák's multiple comparisons test.
