# Supplementary material for: Encoded metabolic remodeling amplifies drug resistance in Mycobacterium tuberculosis
Source: PLoS Pathog. 2026 Aug 14;22(8):e1014237. doi: 10.1371/journal.ppat.1014237 (PMC13497261; doi:10.1371/journal.ppat.1014237)
Supplement: Supplementary file 9 [file ppat.1014237.s009.pdf]

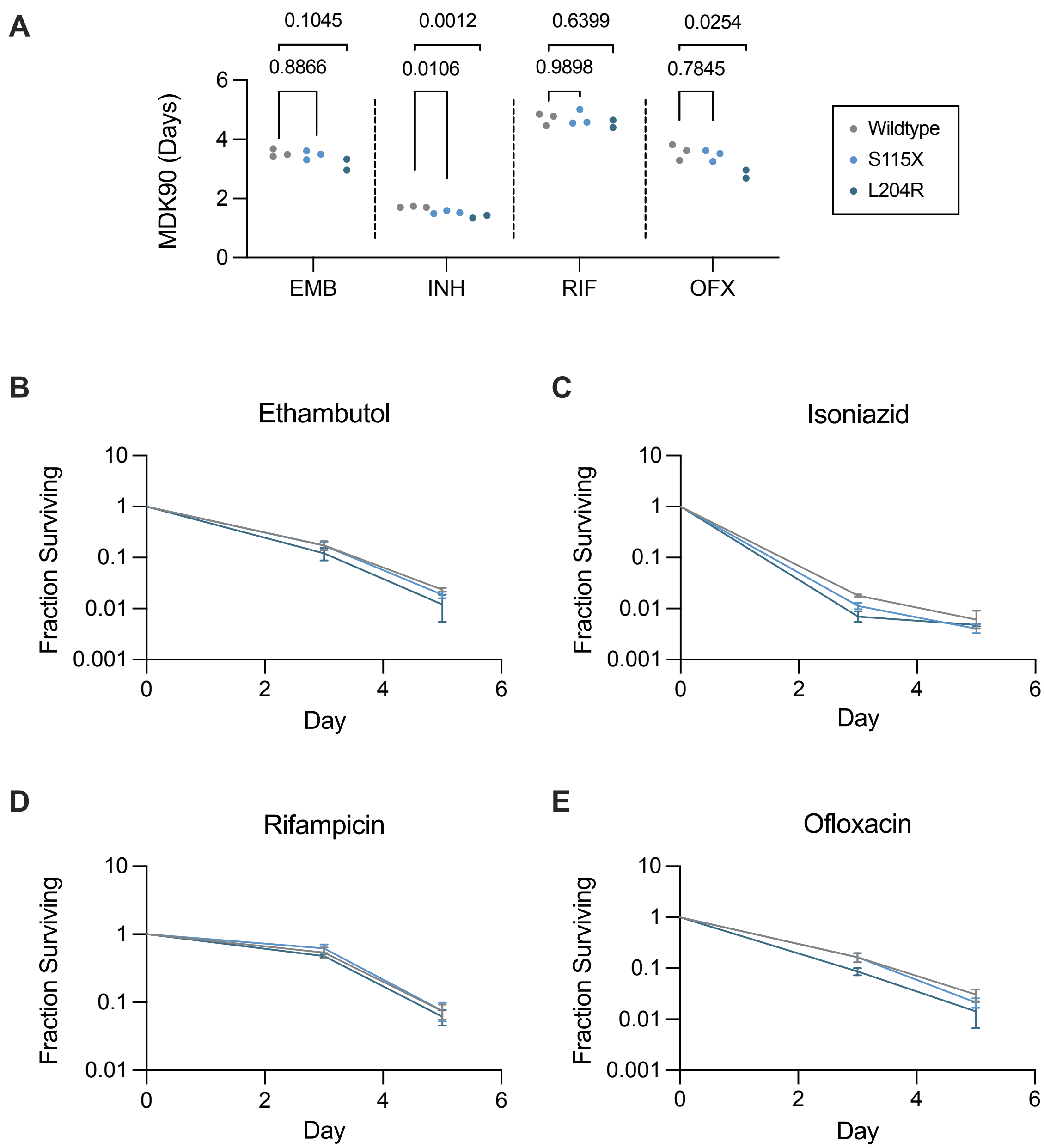

S9 Fig: Kill curve assay. (A) Minimum duration to 90% cell death (MDK90) for wildtype and *idsA2* mutant strains in four tuberculosis antibiotics: ethambutol (EMB), isoniazid (INH), rifampicin (RIF), and ofloxacin (OFX). The concentration of each drug was 55, 3, 0.6, and 34 µg/mL. MDK90 was calculated using a semilog curve equation determined using the line segment through which Fraction Surviving = 0.1 fell. Differences between each mutant and wildtype for each drug were tested by ordinary one-way ANOVA with Dunnett's multiple comparisons test. (B-F) Fraction of surviving bacteria over time calculated from colony enumeration.
