# Supplementary material for: Encoded metabolic remodeling amplifies drug resistance in Mycobacterium tuberculosis
Source: PLoS Pathog. 2026 Aug 14;22(8):e1014237. doi: 10.1371/journal.ppat.1014237 (PMC13497261; doi:10.1371/journal.ppat.1014237)
Supplement: Supplementary file 10 [file ppat.1014237.s010.pdf]

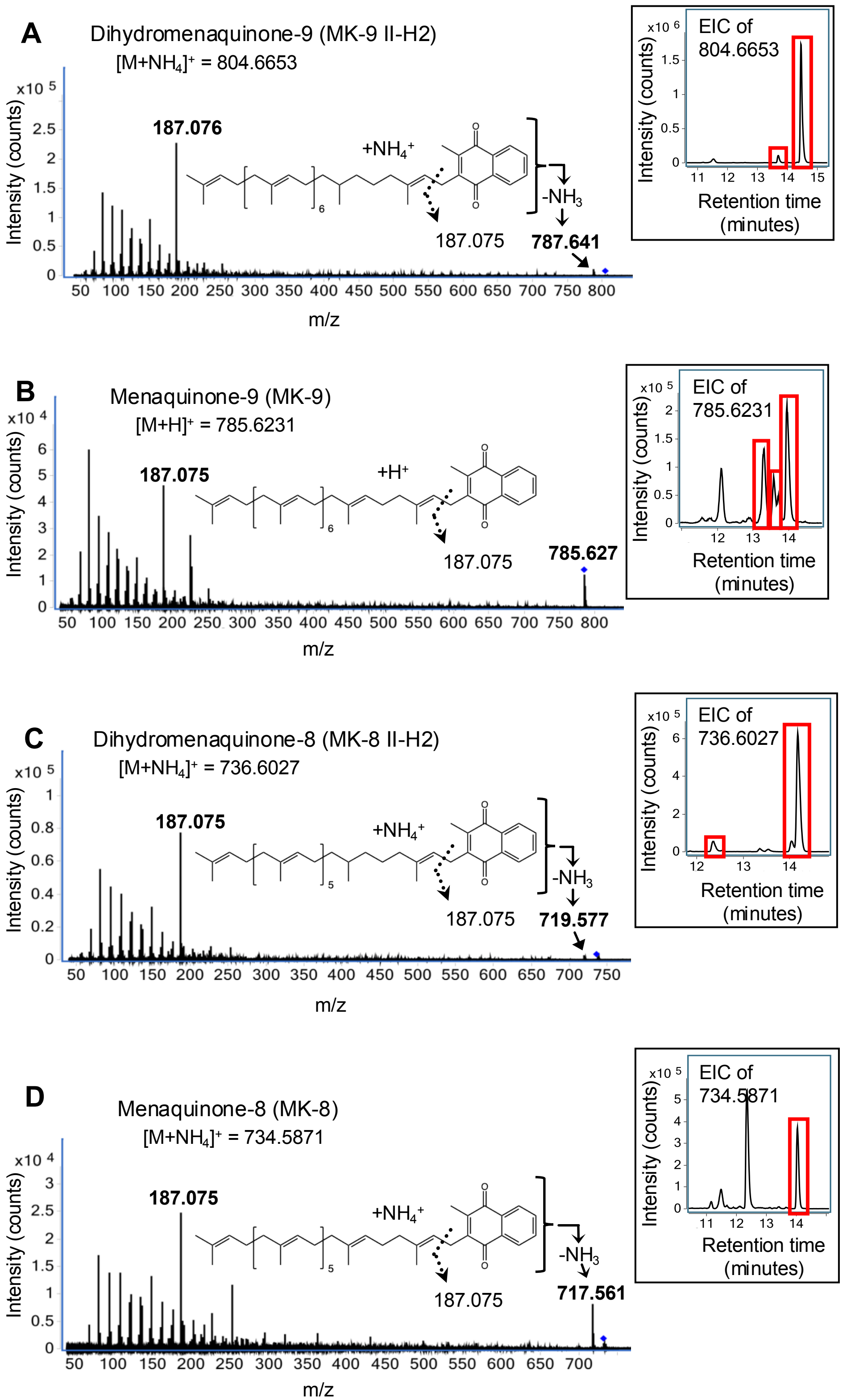

S10 Fig: Representative chromatograms and collision spectra for menaquinone species. Red boxes on extracted ion chromatograms (EICs) indicate peaks with lipid identity confirmed by CID-MS via the highlighted fragments. For each lipid, displayed prenyl stereochemistry and position of saturated groups is assumed from literature patterns and may differ across chromatographic peaks. Blue diamonds in spectra indicate the lowest targeted m/z value for CID-MS.
