# Supplementary material for: Encoded metabolic remodeling amplifies drug resistance in Mycobacterium tuberculosis
Source: PLoS Pathog. 2026 Aug 14;22(8):e1014237. doi: 10.1371/journal.ppat.1014237 (PMC13497261; doi:10.1371/journal.ppat.1014237)
Supplement: Supplementary file 11 [file ppat.1014237.s011.pdf]

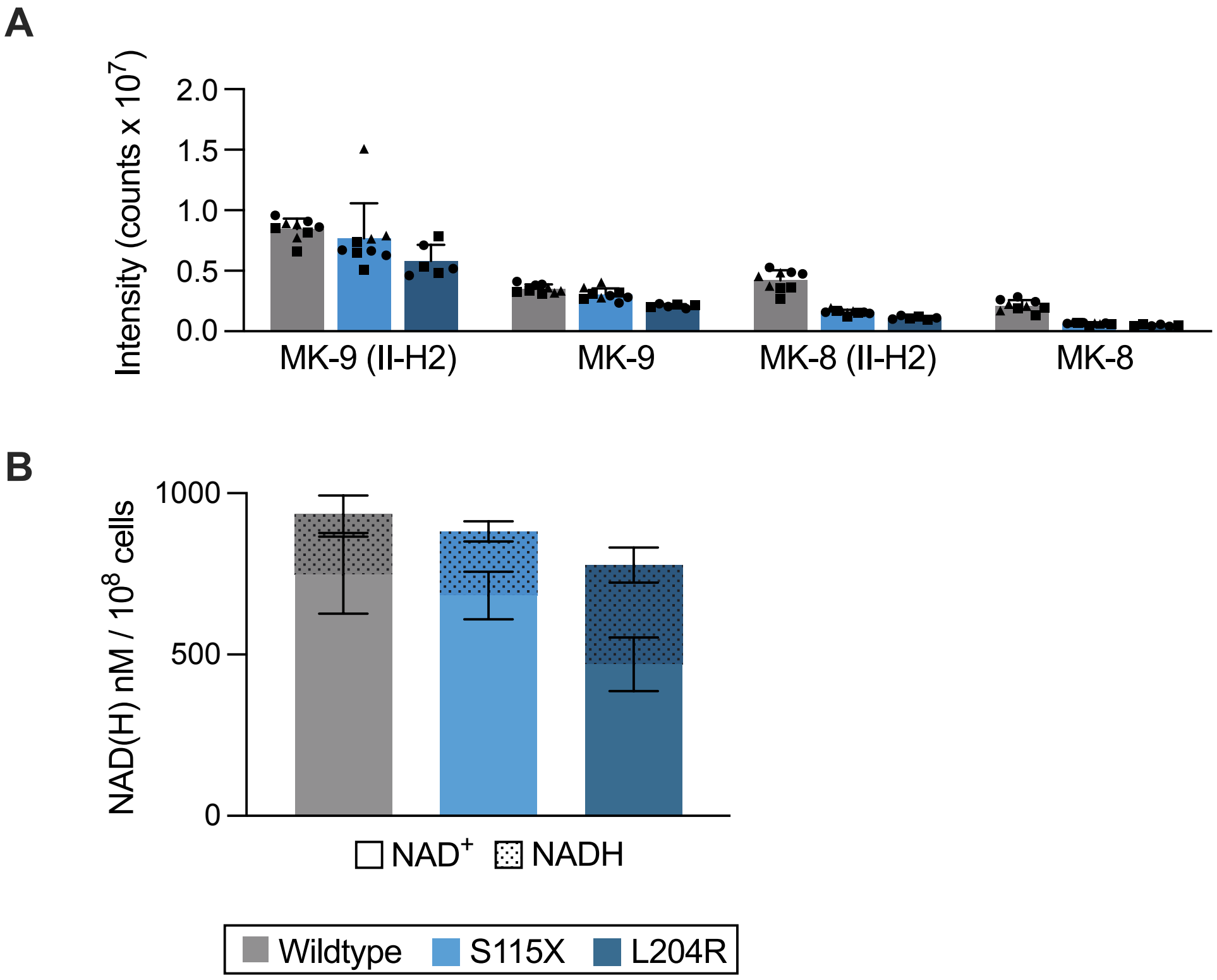

S11 Fig: Supporting data for menaquinone and redox analysis. (A) Intensity measured in arbitrary units (counts) for measured menaquinone species. (B) The concentrations of NAD^+ and NADH as measured by fluorometric assay. Data combines two independent experiments.
