# Supplementary material for: Encoded metabolic remodeling amplifies drug resistance in Mycobacterium tuberculosis
Source: PLoS Pathog. 2026 Aug 14;22(8):e1014237. doi: 10.1371/journal.ppat.1014237 (PMC13497261; doi:10.1371/journal.ppat.1014237)
Supplement: Supplementary file 12 [file ppat.1014237.s012.pdf]

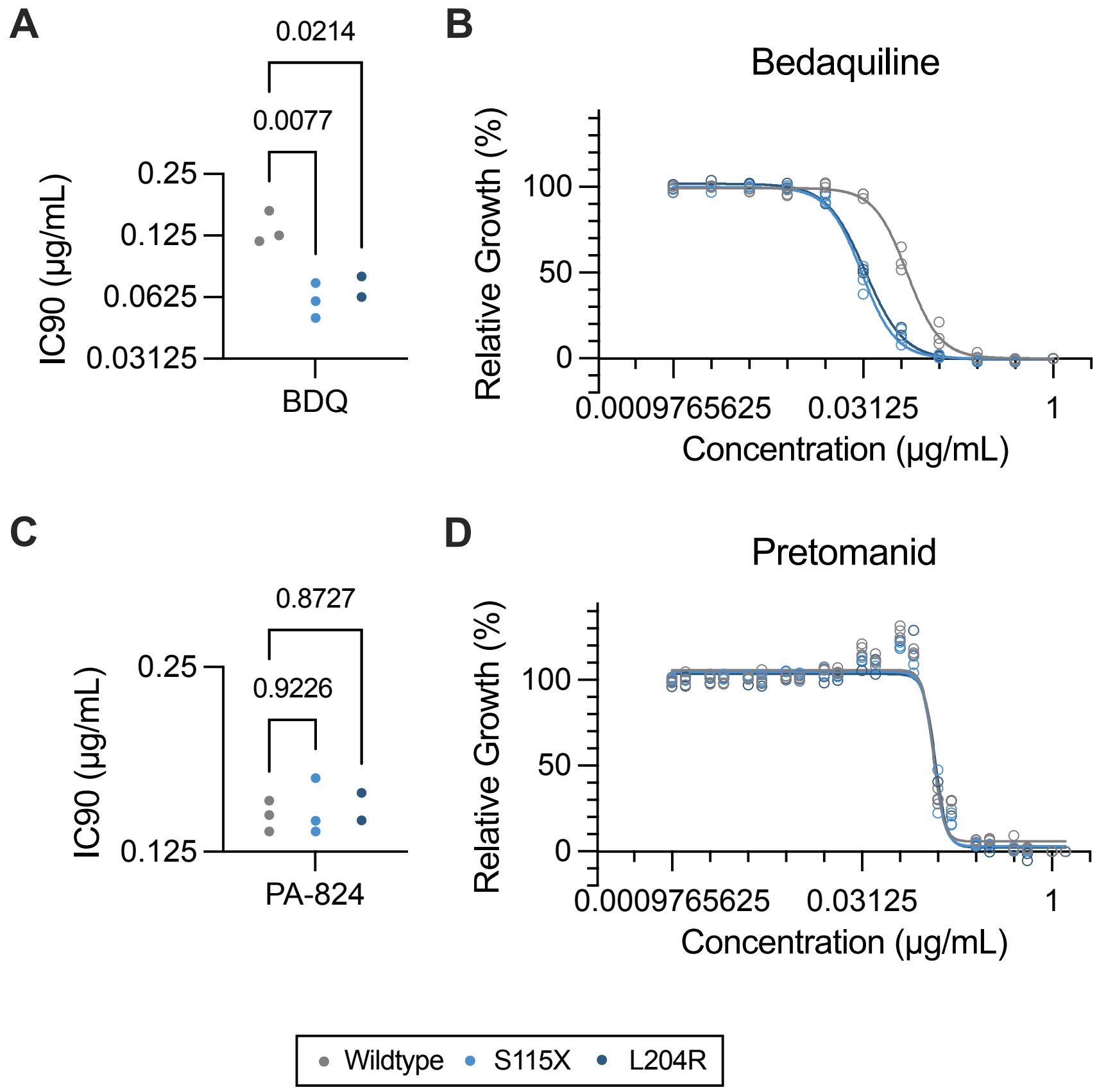

S12 Fig: IC90 statistics and nonlinear regression curves for second-line antibiotics. (A,C) Plots of IC90 values for each strain separated by drug: bedaquiline (BDQ) and pretomanid (PA-824). Differences between each mutant and wildtype were tested by performing ordinary one-way ANOVA with Dunnett's multiple comparisons test. (B,D) Nonlinear regression curves of growth in varying concentrations of antibiotics. Points each represents the average of two (wildtype) to three technical replicates for each clone replicate. For BDQ, the plot is representative of two experiments. For PA-824, points come from two independent experiments.
