# Supplementary material for: Encoded metabolic remodeling amplifies drug resistance in Mycobacterium tuberculosis
Source: PLoS Pathog. 2026 Aug 14;22(8):e1014237. doi: 10.1371/journal.ppat.1014237 (PMC13497261; doi:10.1371/journal.ppat.1014237)
Supplement: Supplementary file 13 [file ppat.1014237.s013.pdf]

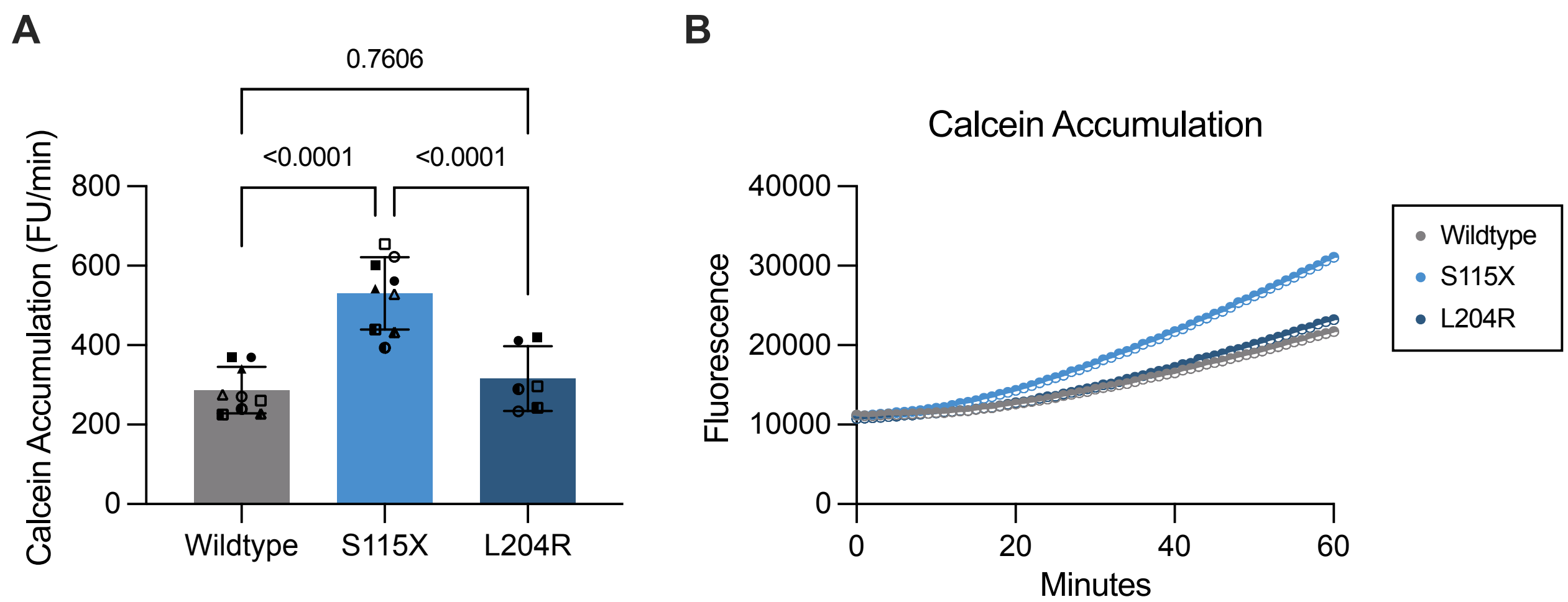

S13 Fig: Calcein accumulation assay. (A) Rate of calcein accumulation following 20 minutes of equilibration, as measured by a simple linear regression on the calcein accumulation curve. Data combines three experiments, indicated by dot fill while dot shape indicates clone replicates. Differences between each strain are measured by ordinary one-way ANOVA with Tukey's multiple comparisons test. (B) Representative curves of calcein accumulation over time.
