# Supplementary material for: Encoded metabolic remodeling amplifies drug resistance in Mycobacterium tuberculosis
Source: PLoS Pathog. 2026 Aug 14;22(8):e1014237. doi: 10.1371/journal.ppat.1014237 (PMC13497261; doi:10.1371/journal.ppat.1014237)
Supplement: Supplementary file 14 [file ppat.1014237.s014.pdf]

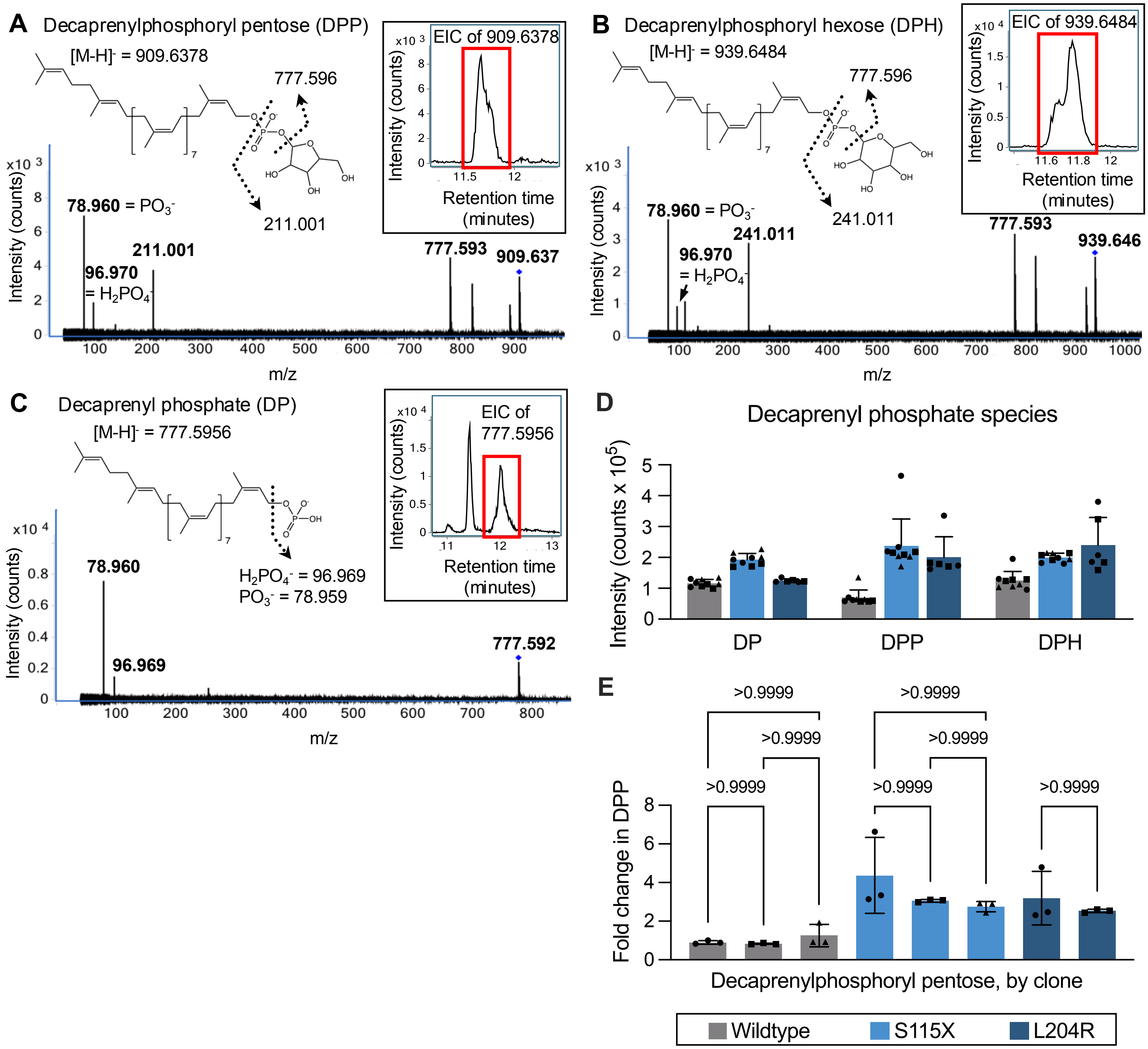

S14 Fig: Supporting data for polyprenyl analysis. (A-C) Representative chromatograms and collision spectra for decaprenyl phospholipids. Red boxes on EICs indicate peaks with lipid identity confirmed by CID-MS via the highlighted fragments. For each lipid, displayed prenyl stereochemistry and position of saturated groups is assumed from literature patterns and may differ across chromatographic peaks. Blue diamonds in spectra indicate the lowest targeted m/z value for CID-MS. (D) Intensity measured in arbitrary units (counts) for measured decaprenyl phosphate species. (E) Fold change in DPP levels as measured by LCMS where each bar is a clone, and dots are culture replicates. Different shapes indicate different clone replicates corresponding with Fig 5C. Differences in DPP levels determined by Kruskal-Wallis test with Dunn's multiple comparisons test.
