# Supplementary material for: Encoded metabolic remodeling amplifies drug resistance in Mycobacterium tuberculosis
Source: PLoS Pathog. 2026 Aug 14;22(8):e1014237. doi: 10.1371/journal.ppat.1014237 (PMC13497261; doi:10.1371/journal.ppat.1014237)
Supplement: Supplementary file 15 [file ppat.1014237.s015.pdf]

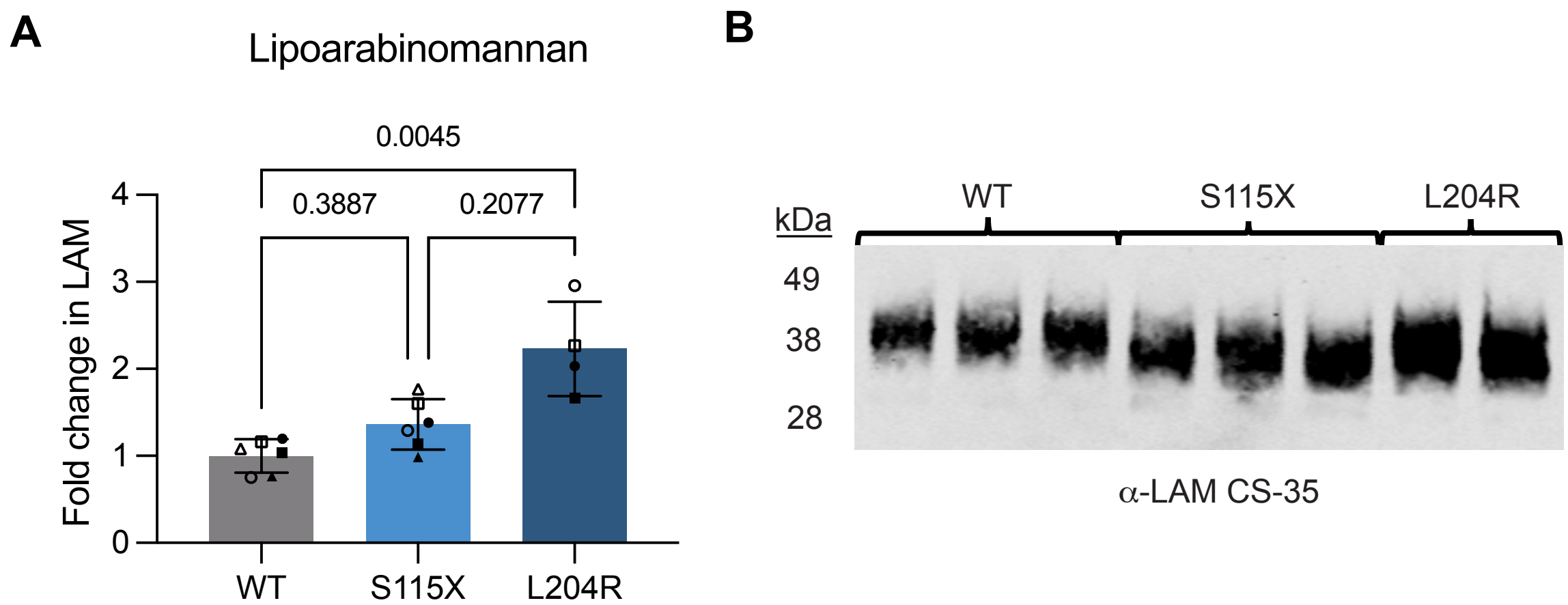

S15 Fig: Lipoarabinomannan (LAM) measurement by western blot. (A) Fold change in LAM levels as measured by western blot with anti-LAM CS-35 antibody. Data from two experiments indicated by dot fill while dot shapes indicate clone replicates. Differences in LAM levels determined by Kruskal-Wallis test with Dunn's multiple comparisons test. (B) Representative western blot.
