# Supplementary material for: Encoded metabolic remodeling amplifies drug resistance in Mycobacterium tuberculosis
Source: PLoS Pathog. 2026 Aug 14;22(8):e1014237. doi: 10.1371/journal.ppat.1014237 (PMC13497261; doi:10.1371/journal.ppat.1014237)
Supplement: Supplementary file 16 [file ppat.1014237.s016.pdf]

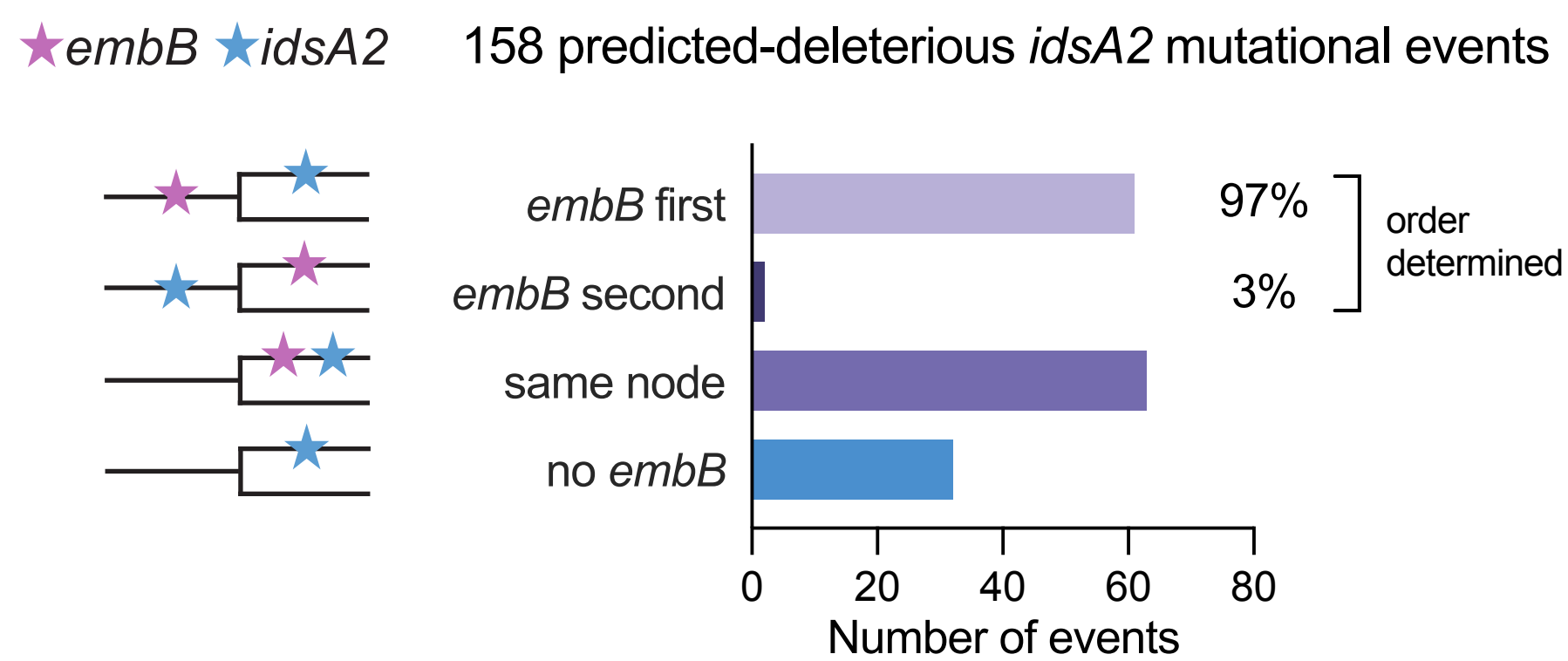

S16 Fig: Ordering of predicted-deleterious *idsA2* mutational events. Ordering of 158 predicted-deleterious idsA2 mutational events within Lineage 4 of the 55K dataset.
