# Supplementary material for: Encoded metabolic remodeling amplifies drug resistance in Mycobacterium tuberculosis
Source: PLoS Pathog. 2026 Aug 14;22(8):e1014237. doi: 10.1371/journal.ppat.1014237 (PMC13497261; doi:10.1371/journal.ppat.1014237)
Supplement: Supplementary file 17 [file ppat.1014237.s017.pdf]

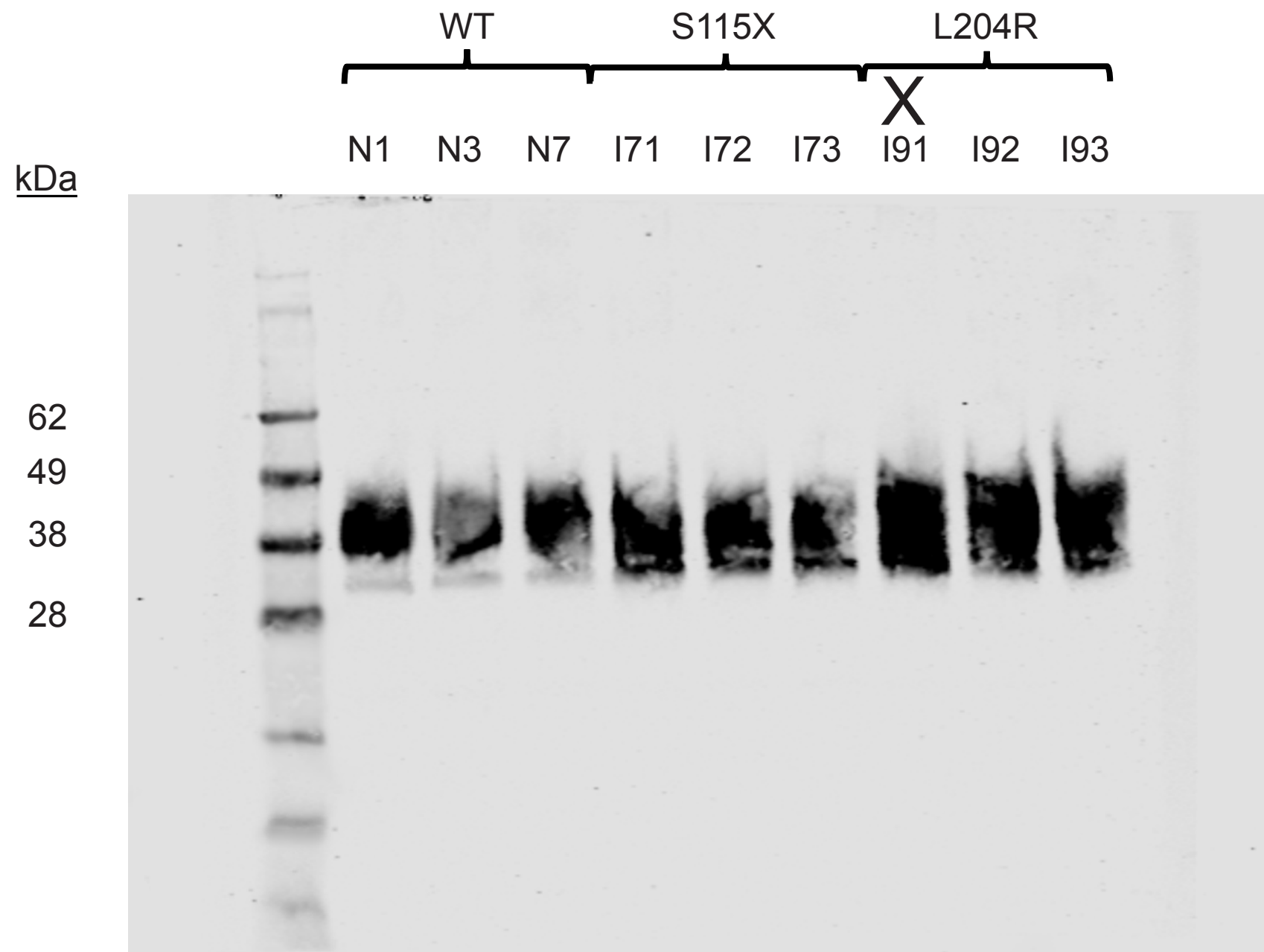

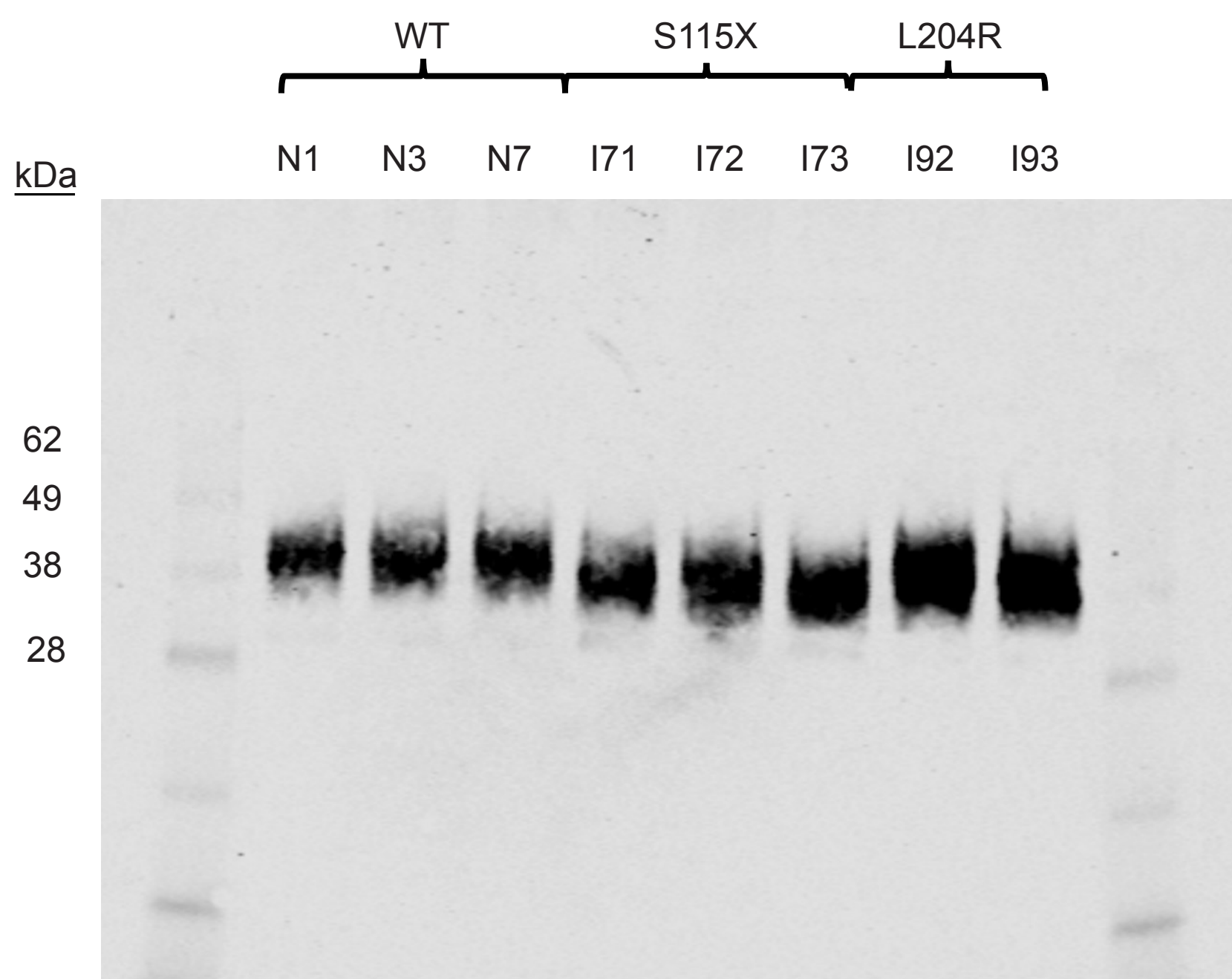

Raw images for LAM Western Blots. Two western blots were performed. Ladder is SeeBlue Plus2 Pre-stained Protein Standard. Primary antibody is Monoclonal Anti-*Mycobacterium tuberculosis* LAM antibody, Clone CS-35. Secondary antibody is IRDye 800CW Goat anti-Mouse IgG Secondary Antibody. Images were taken on a LI-COR Odyssey CLx Imager and processed and analyzed using Empiria Studio. Data from both experiments is reported in S15A Fig, and the second blot is shown as a representative image in S15B Fig.
