# Supplementary material for: Encoded metabolic remodeling amplifies drug resistance in Mycobacterium tuberculosis
Source: PLoS Pathog. 2026 Aug 14;22(8):e1014237. doi: 10.1371/journal.ppat.1014237 (PMC13497261; doi:10.1371/journal.ppat.1014237)
Supplement: Supplementary file 18 [file ppat.1014237.s024.docx]

Dear Dr. Behr,

Thank you for your consideration of our manuscript. We would also like to sincerely thank the reviewers for their time and effort. We have revised the manuscript to address each of the comments and believe that the manuscript is substantially improved. We have responded to each question and comment individually below, sorted by reviewer. We have included updated line numbers for convenience; these numbers correspond only to the marked-up copy of the manuscript. Again, thank you for your time and effort.

Sincerely,

Abigail Frey

**Reviewer 1:**

We thank Reviewer 1 for their detailed feedback helping us to improve the clarity of this manuscript. We have responded to each comment and question individually below.

1. Line 121: “SIFT-predicted deleterious missense mutation” Since SIFT is arguably not a widely familiar tool (vs. e.g., something like BLAST), suggest removing, briefly describing, or referring to methods for clarity.

We removed the term “SIFT” and provided clarifying description on line 127 instead:

“missense mutation computationally predicted to be deleterious based on sequence homology and the physical properties of amino acids.”

2. Line 131: which --> that. Please check overall for correct use of these relative pronouns.

We edited the indicated sentence and made several additional alterations to our use of relative pronouns throughout.

3. Line 136, Fig. 2 and Line 167, Fig. 3. There appears to be a discrepancy in the RIF sensitivity of idsA2 mutational events as determined from the CRyPTIC dataset (more resistant; Fig. 2) vs. from individual mutants (more sensitive; Fig. 3A, D). Could the authors please comment?

We thank Reviewer 1 for pointing out this apparent discrepancy. In the CRyPTIC dataset, 40/72 strains with *idsA2* mutations also have mutations in the target of rifampicin, *rpoB.* This can be seen in the plot below, which shows *idsA2* mutant strains plotted by rifampicin MIC and with strains with *rpoB* mutations shown in pink. Consequently, the association between *idsA2* mutations and increased rifampicin MIC in the CRyPTIC dataset is due to the enrichment of *rpoB* mutations among *idsA2* mutant strains. The co-occurrence between *idsA2* mutations and *rpoB* mutations causing high level rifampicin resistance in clinical strains is consistent with the genetic linkage that characterizes drug resistance variants in Mtb. On the other hand, since there are no rifampicin resistance mutations in the isogenic strains, we can detect smaller differences attributed to the presence of an *idsA2* mutation.

To clarify this point, we added Fig S6, which includes the panel above, and adjusted the text starting at line 147 in the Results stating:

“Due to the concurrent use of these drugs for treatment of tuberculosis, mutations altering drug susceptibility are genetically linked (S6 Fig). Therefore, these data demonstrate that *idsA2* mutations are associated with first-line drug resistance mutations, but not whether *idsA2* mutations themselves shift the MIC of any of these drugs.”

Finally, we addressed this discrepancy and the reason behind it in more detail in the Discussion starting at line 396:

“This work demonstrates the complex ramifications of metabolic rewiring on drug susceptibility and the importance of epistatic interactions on the presentation of those effects. For example, we observed that *idsA2* mutations are sufficient to increase the sensitivity of rifampicin in isogenic strains. However, in the clinical strains assayed in the CRyPTIC dataset, *idsA2* mutations often co-occur with mutations in *rpoB*, which cause high-level rifampicin resistance, seemingly abolishing any enhanced susceptibility that the *idsA2* variant may have conferred.”

4. Related, what assay was used to the determine MIC in the CRyPTIC dataset? Could any differences explain the RIF discrepancy?

The CRyPTIC consortium used custom, pre-prepared 96-well 7H9 broth microdilution plates, with multiple forms of readout depending on the laboratory, all of which were visible-growth based (The CRyPTIC Consortium, 2022). Reviewer 1 makes a good point that our technique and that of the CRyPTIC Consortium are likely to produce slightly differing results. However, the association between increased rifampicin MIC and *idsA2* mutations in the CRyPTIC consortium data can be explained based on the co-occurrence drug resistance mutations as described above.

5. This study used Alamar Blue, which reports on respiration but may not report reliably on viability depending on metabolic state. Given that idsA2 mutations affect energy/metabolic status, did the authors determine MIC, especially for EMB, by an independent assay?

We agree with Reviewer 1 that AlamarBlue’s dependance on metabolic state is a limitation of the method, especially given the changes to NAD^+^/NADH ratio we observe in the *idsA2* mutant strains. To verify that *idsA2* mutant strains continue to grow when subjected to the wildtype MICs of ethambutol and isoniazid we performed a growth-based competition assay. In brief, we combined wildtype strains with each *idsA2* mutant and grew them for 5 days in no drug, or ethambutol or isoniazid at the wildtype IC90 calculated using the AlamarBlue assay. We then extracted gDNA and used Sanger sequencing to assess the relative abundance of the *idsA2* wildtype vs mutant strains. Consistent with the AlamarBlue data, both *idsA2* mutants outcompete the wildtype strain in ethambutol and isoniazid, while in the no drug control the mutant proportion decreases relative to input, consistent with the growth defect we observed in axenic culture.

We describe this result at line 178 as follows:

“We validated these results in a growth-based competition assay, which demonstrated that the *idsA2* mutant strains have a significant fitness advantage over wildtype Mtb in ethambutol and isoniazid at drug concentrations equivalent to the IC90s of the wildtype strain.”

Additionally, the full description of the competition assay method can be found starting at line 635.

6. Line 196: “concentrations in metabolite [] from wildtype and…” Word appears to be missing at brackets? Metabolite extracts? Related also to Line 214, which refers to analysis of “whole-cell lysates”. Methods report lysis/extraction in acetonitrile:MeOH:water. Suggest adding/modifying wording for clarity and consistency throughout.

We thank Reviewer 1 for the opportunity to improve this wording. We have made the clarifications at what are now lines 212 which now states

“Concentrations of C_5_ (DMAPP) and *E-*C_20_ from wildtype and S115X mutant Mtb as measured by LCMS against standards added quantitatively.”

And at line 231 stating:

“we lysed the *idsA2* nonsense mutant (S115X) and wildtype strains in acetonitrile, methanol, and water and detected metabolites using liquid chromatography mass spectrometry (LCMS).”

7. Line 258: Please clarify briefly for non-expert readers why DPP is a combined measure of DPA and DPR and how later conclusions based on inference of DPA levels is valid.

We appreciate Reviewer 1 bringing this to our attention. We now describe how DPP is a combined measure and yet a reasonable readout of DPA starting at line 282. The relevant paragraph is inserted below.

“Notably, one decaprenyl phosphate species with significantly increased levels in the *idsA2* mutant strains was decaprenylphosphoryl pentose (DPP) (Figs 5C and S14). Previous work has found that mutation or upregulation of the decaprenylphosphoryl-5-phosphoribose (DPPR) synthase, encoded by *ubiA*, also increased both DPP levels and ethambutol MIC [42–44]. Since DPP is the combined measure of decaprenylphosphoryl arabinose (DPA) – the carrier molecule for arabinose [45,46] – and the precursor to DPA, decaprenylphosphoryl ribose (DPR), it was proposed that the increased quantity of DPA allowed arabinose to outcompete ethambutol for binding to its drug target, the arabinosyltransferase EmbB [44,47,48]. In support of this model, we found that in addition to increased levels of DPP, *idsA2* mutants trended towards higher levels of lipoarabinomannan (LAM), reflecting an increase in product abundance downstream of DPA synthesis, and suggesting that proportional changes in DPP are a reasonable readout to estimate proportional changes in DPA levels (S15 Fig). Together, our genomic, functional, and metabolic data support a model in which *idsA2* variants increase the inhibitory concentration of ethambutol through increased production of DPA.”

We have also added a technical note on this combined measurement starting at line 752 in the Methods.

8. Lines 290-292: The methodology of generating the double mutants is somewhat confusingly worded, as idsA2 mutations were generated by recombination, but the embB mutation by selection and, importantly, sequencing. Please modify for clarity.

We are grateful for the opportunity to make this method clearer and have adjusted the segment starting at line 321 as follows:

“To experimentally test the effects of *idsA2* variants in combination with *embB* mutations, we selected for ethambutol resistance mutations in both the *idsA2* mutant and control strains. Representative strains from each *idsA2* strain background were grown on ethambutol, candidate colonies were whole-genome sequenced, and two independent clones with the ethambutol resistance-associated *embB* Q497R mutation from each strain background were selected [47]*.*”

9. Lines 293-296: Would resistance-conferring mutations be expected to increase MICs by a fixed concentration amount, or by fold change? On both backgrounds, addition of idsA2 mutations increase MIC by 2-fold. Please comment and possibly modify the “however” in line 295 accordingly.

We appreciate the interesting question from Reviewer 1. We think this question is best addressed using data from Figure 6 from the CRyPTIC Consortium’s paper “Epidemiological cut-off values for a 96-well broth microdilution plate for high-throughput research antibiotic susceptibility testing of M. tuberculosis” (The CRyPTIC Consortium, 2022). In this figure, the distribution of MICs across clinical strains collected by the CRyPTIC Consortium is shown for 8 drugs, and the number of strains with certain drug resistance variants is indicated. Where sufficient data does exist, such as for the contribution of *katG* S315T to isoniazid resistance, we see that the variant does not have a fixed concentration effect on isoniazid MIC. However, the more general assessment of these models is hindered by a variety of factors, including simple technical considerations such as dynamic range of the assay. For example, nearly all *rpoB* S450L mutations fall in the >8 µg/mL bin, so it is not clear what the true MICs of the strains with this variant are. Overall, this question illustrates the amount of opportunity for future work to understand the effect of drug resistance variants in different strain backgrounds.

To address this point in the text, we removed the indicated “however”, now line 331, and emphasized the two-fold shift conferred by *idsA2* regardless of strain background. The section now states:

“In the absence of additional ethambutol-resistance mutations, *idsA2* mutations increased the ethambutol IC90 by 0.5 µg/mL, resulting in an IC90 of 1 µg/mL; this is a two-fold shift from baseline (Fig 3 and 6B-C). In the presence of *embB* Q497R strain, *idsA2* mutations increased ethambutol IC90 by 4 µg/mL, resulting in an IC90 of 8 µg/mL (Figs 6B-C). Therefore, *idsA2* mutations maintain the ability to shift the inhibitory concentration of ethambutol by two-fold, even when starting at a higher baseline ethambutol IC90.”

10. Related, line 425: “Most notably, idsA2 mutations have a multiplicative effect with embR variants on [EMB] resistance.” (and similar in abstract, line 35) As noted above, they have a multiplicative effect on WT background also. Please modify/clarify accordingly.

We thank Reviewer 1 for the opportunity to improve these statements. We have modified the two sections accordingly now found at line 490:

“Notably, we show that *idsA2* mutations also augment resistance on top of *embB* mutations, elevating resistance to the clinical breakpoint.”

And at line 36:

“Where *idsA2* mutations arise after *embB* mutations, they maintain their multiplicative effect on the inhibitory concentration of ethambutol such that the variants together result in high-level resistance.”

11. Line 336: “presence of… an idsA2 mutation…” Fig. 7A shows analysis for embB mutant, but not idsA2 mutant. Are the data for this conclusion (lower false positive rate with inclusion of idsA2 mutant alone) shown anywhere? Please clarify.

We thank Reviewer 1 for this question. To better visualize this finding, we added panel B (also shown below) to Figure 7. While what is now panel C also shows the *embB+idsA2* mutant strains, and the strain counts can be found in S1 Data, we agree the inclusion of this panel better demonstrates the decrease in the number of false positive strains in *embB*+idsA2 strains, as well as the low abundance of *idsA2* mutants that lead to the interest in all second-step mutations.

12. Line 353: “This pathway remodeling also enables its most clinically relevant effect” The most relevant connection to resistance is the model that increases in DPA compete for ethambutol binding to embB, but this is not directly demonstrated. Suggest modulating wording for correlation vs. causation.

We thank Reviewer 1 for pointing this out. We adjusted the wording of this sentence which is now at line 394:

“This pathway remodeling is also linked to its most clinically relevant effect, amplifying ethambutol resistance after *embB* mutation.”

13. On that note, lines 33-34: “increased production of [DPP], which can compete with ethambutol for binding to arabinosyltransferases” vs. line 262: “it was proposed that… increasing the quantity of DPA allows arabinose to outcompete ethambutol for binding” Is there in fact experimental evidence from any previous study for this competition? If not, suggest modifying abstract accordingly. Also please note and consider resolving the discrepancy/confusion regarding referring to DPP as the actual measure vs. DPA as the specific relevant metabolite (see related comment above).

We appreciate Reviewer 1 catching this detail. In the published study in which arabinose was proposed to outcompete ethambutol for binding to arabinosyltransferases there was no direct evidence for a change in binding kinetics (Safi *et al.* 2018). Instead, the authors showed that overexpression of *ubiA,* in addition to *ubiA* mutation, both increases DPP levels and increases ethambutol MIC. We have adjusted line 35 in the Abstract to instead state:

“This suggests that *idsA2* and *ubiA* mutations share an ethambutol resistance mechanism.”

We have also resolved the issue of DPP being the measure and DPA being the relevant metabolite in the paragraph included under question 7, and by referring to decaprenyl phosphate species when necessary, such as in the lines edited in question 14 below.

14. Line 423: “through the pleiotropic effects of metabolite remodeling” We feel that this wording is reasonable given the data, but confusing in this manuscript due to the previous emphasis in abstract and main text on the inferred contribution of elevated DPA to ethambutol inhibition of the EmbB target. A minor point, but possibly combining these would be most clear—that is, there are pleiotropic effects, of which elevated DPP/DPA provides the most direct connection to EMB mechanism of action. Please consider modifying for consistency and accuracy, with advance thanks for helping especially non-expert readers achieve the clearest possible sense of the conclusions.

We thank Reviewer 1 for the suggestion. We edited line 487 onward based on this suggestion. They now read:

“In this work, we show that *idsA2* variants have pleiotropic effects on the cell, of which the elevated abundance of decaprenyl phosphate species provides the most direct connection to the ability of *idsA2* mutants to grow in higher ethambutol concentrations.”

**Reviewer 2:**

We thank Reviewer 2 for their helpful suggestions regarding additional data to include in this manuscript. We have responded to each comment and question individually below.

1. The abstract should make it clear that the impact of idsA2 mutation(s) on EMB resistance appears restricted to strains within lineage 4. Similarly, the increase in MIC (or more correctly IC90) is relatively small and in the order of 2-fold. Personally, I think the abstract oversells the case for idsA2, particularly with statements such as “Together, this work defines idsA2 as an ethambutol resistance gene”. By itself, idsA2 does not appear to confer EMB resistance. Please temper this statement to more accurately reflect the reality.

We strive to accurately represent our data and thank Reviewer 2 for the opportunity to improve the abstract. We note the Lineage 4 specificity in line 28, specify the 2-fold observation in the inhibitory concentration of ethambutol at lines 31-32, and temper our conclusion at line 41. The abstract is included below for your convenience:

“Antibiotic pressure causes pathogens to evolve many forms of altered drug susceptibility. In addition to target or activator mutations conferring canonical drug resistance, mutations can serve as steppingstones to or enhancers of resistance. In clinical strains of *Mycobacterium tuberculosis* (Mtb), we find that *idsA2,* which encodes an isoprenyl pyrophosphate synthase involved in the synthesis of precursors for essential components of the cell wall and electron transport chain, is undergoing diversifying selection in Lineage 4, and that these mutations are associated with the acquisition of first-line antibiotic resistance. By engineering isogenic Mtb strains that express clinically prevalent variants of *idsA2*, we show that clinical variants alter the inhibitory concentrations of first-line drugs, most significantly increasing the inhibitory concentration of ethambutol by two-fold. Targeted lipid analyses reveal that disrupting IdsA2 function redirects limited resources in the isoprenoid synthesis pathway, leading to increased production of decaprenyl phosphate species. This suggests that *idsA2* and *ubiA* mutations share an ethambutol resistance mechanism. Where *idsA2* mutations arise after *embB* mutations, they maintain their multiplicative effect on the inhibitory concentration of ethambutol such that the variants together result in high-level resistance. As a result, identification of *idsA2* mutations can be utilized to improve the specificity of genotypic ethambutol susceptibility testing. Together, this work shows how *idsA2* mutations remodel bacterial metabolism and augment ethambutol resistance.”

2. Are the frequencies of EMB-R strains comparable between the 4 Mtb lineages that make up the 55K database? Please comment in the text on this.

We thank Reviewer 2 for bringing this up. The rate of ethambutol drug resistance in the 55K dataset varies between lineages, with Lineage 2 having 45% of strains (7329/16270) containing a WHO-verified ethambutol resistance mutation, compared to 15% of Lineage 4 strains (4341/28817), 13% of Lineage 3 strains (761/6015), and 8% of Lineage 1 strains (321/4120). The increased rate of drug resistance in Lineage 2 is consistent with the literature (Goig *et al.* 2025, Torres *et al.* 2021). We now note the differing rates of ethambutol resistance as a potential explanation for the lineage-specific selection of *idsA2* starting at line 457:

“It should also be noted that, consistent with the literature [1,57], in our datasets there is a higher rate of *embB* mutations conferring ethambutol resistance in Lineage 2 strains (45% in the 55K dataset) compared to the other lineages (8-15% in the 55K dataset). This could contribute to the observed lineage-specific selective pressure on *idsA2* or could be an additional example of epistasis*.*”

3. Regarding the CRyPTIC dataset, 19 lineage 4 strains seem to have a predicted deleterious idsA2 mutation (Figure S4). How many of these also have a canonical mutation in embB? Is it possible to indicate which strains have an embB mutation on the relevant panel of figure S4?

We appreciate this question and suggestion from Reviewer 2. Figure S4 shows the differences in MIC attributed to *idsA2* mutational events relative to the nearest neighbor strain(s). Therefore, to address this question, we created a new figure showing the distribution of MICs across Lineage 4 strains from the CRyPTIC dataset with predicted-deleterious *idsA2* mutations. We colored the strains which also share *embB* mutations in pink. Consistent with the strong association between predicted-deleterious *idsA2* mutations and drug resistance, we found that 20/23 mutant strains have *embB* mutations. 19/20 of those *embB* mutations are variants associated with ethambutol resistance according to the WHO. (The one *embB* mutant that does not have a WHO-associated variant has an MIC of 4 µg/mL and is not the strain which has an MIC of 0.5 µg/mL.)

The panel above is included in what is now Figure S6. We performed this analysis for both our initial *idsA2* variant calling settings as well as predicted-deleterious variants and for all three first line drugs to best illustrate the association between the variants and drug resistance.

4. Lines 133-134: “We calculated the average difference in MICs between Lineage 4 strains with idsA2 mutations ….”. What is the result if this same calculation is performed for lineage 1-3 strains?

We thank Reviewer 2 for this question. To assess whether the statistically significant associations found between *idsA2* mutational events and increases in the MICs of rifampicin, isoniazid, and ethambutol were also present across Lineages 1, 2, and 3, we repeated this analysis for those lineages. After multiple test correction, we did not find any statistically significant associations. This result can be seen to the right. We note that the average differences in MIC for isoniazid and ethambutol in Lineage 3 were higher than those in Lineage 4. The lack of statistically significant associations may be attributed to the relatively lower number of *idsA2* mutational events in Lineage 3 (6-7 after MIC quality control) compared to Lineage 4 (36-44 after MIC quality control). It would be interesting to repeat this analysis when more whole genome sequencing has been performed on Lineage 3 strains.

This data is included in S5 Fig and described as follows on lines 145-147:

“We did not identify a statistically significant association between *idsA2* mutational events and increases in the MICs of rifampicin, isoniazid, or ethambutol in Lineages 1, 2, or 3 (S5 Fig).”

5. Unfortunately, aside from the standard fall-back H37Rv lab strain, the authors do not seem to have carried out the in vitro idsA2 recombineering with a non-lineage 4 strain to examine its effect on isoprene metabolism and/ or the EMB IC90. A lack of an effect on isoprene metabolism (with a corresponding lack of EMB MIC shift) would have gone a long way towards supporting the author’s suggestion that the effect is truly lineage specific.

We agree with Reviewer 2 that we have not established whether the effect of *idsA2* mutations (meaning the presence of isoprenoid remodeling or ethambutol IC90 change) is restricted to Lineage 4. Using population data, we demonstrate that selection on *idsA2* is largely Lineage 4-specific, but this could be due to either the effect of *idsA2* mutation being restricted to Lineage 4 or the ability of *idsA2* to mutate being restricted to Lineage 4, perhaps because an epistatic mutation changes the conditional essentiality of *idsA2*. We agree this would be an interesting question for future work and have clarified this remaining work starting at line 462 in the Discussion:

“Overall, while we have established that there is an increased rate of *idsA2* mutation in Lineage 4, it remains to be tested whether an *idsA2* mutation introduced to a strain from another lineage would recapitulate the phenotypes we observed in Lineage 4.”

6. Lines 295-297: In my opinion, this material is written in a somewhat misleading/deceptive manner. The relative increase in IC90 due to the idsA2 mutations is still only 2-fold, regardless of whether they co-occur with a canonical embB mutation(s) responsible for conferring EMB-R. In fact, what has caused the 8-fold shift in IC90 is the embB mutation, ie. 0.5 ug/ml to 4ug ug/ml in the absence of the idsA2 SNP; 1ug/ml to 8ug/ml in the presence of the idsA2 SNP.

We agree with Reviewer 2 that *idsA2* mutations are responsible for a 2-fold shift regardless of the strain backgrounds tested and had no intention for our statements to be misleading. To more clearly state the role of *idsA2* mutations in this section, we emphasize that *idsA2* mutations confer a two-fold shift in both strain backgrounds, while including the quantitative measurements so that the reader understands that concentration increase when starting from different backgrounds varies. This can now be found starting at line 328 and below.

“In the absence of additional ethambutol-resistance mutations, *idsA2* mutations increased the ethambutol IC90 by 0.5 µg/mL, resulting in an IC90 of 1 µg/mL; this is a two-fold shift from baseline (Fig 3 and 6B-C). In the presence of *embB* Q497R strain, *idsA2* mutations increased ethambutol IC90 by 4 µg/mL, resulting in an IC90 of 8 µg/mL (Figs 6B-C). Therefore, *idsA2* mutations maintained the ability to shift the inhibitory concentration of ethambutol by two-fold, even when starting at a higher baseline ethambutol IC90.”

**Reviewer 3:**

We thank Reviewer 3 for their careful review of our genomics methods and the additional feedback. We have responded to each comment and question individually below.

1. The GitHub repository is not currently active to review the details of the nearest-neighbor approach described on lines 477-498. I did not quite understand how exactly this analysis is performed. If the analysis is comparing the MICs of a subtree subtended by a node defined as having an ancestral state with an idsA2 mutation to the nearest neighbor – is there any check to ensure that the nearest neighbor subtree (however defined) does not also contain an idsA2 mutation somewhere within it? Apologies if I have misunderstood the approach, perhaps it can be clarified in the methods text. Could a phylogenetic comparative method also be useful here to address the question?

We are grateful to Reviewer 3 for the review of our nearest neighbor analysis. We have activated the GitHub repository and apologize for any inconvenience this delay caused. The provided description is correct; this analysis is comparing the average MICs of strains under a node with an *idsA2* mutation, with the average MICs of the strains defined as the nearest neighbor(s). Before deciding on this approach, we assessed whether the presence of *idsA2* mutant strains among the nearest neighbor(s) would significantly impact the results. Before filtering out strains without high quality MIC values for a specific drug, we identified 61 *idsA2* mutational events on Lineage 4 of the CRyPTIC tree, and out of those 61 events, there are only three cases in which an *idsA2* mutation appears in a nearest neighbor. In two of these cases, the nearest neighbor is also a node with an *idsA2* mutation; therefore, these two events effectively cancel each other out. In the third case, there is a single *idsA2* mutant strain present in the largest of the nearest neighbor subtrees, which contains 239 strains; therefore, the contribution of the *idsA2* mutant strain is minimal. Based on this information, in order to perform a permutation test with the same procedure as was performed for the calculation on *idsA2* events, we allow these cases to remain in the dataset. In total, we agree with Reviewer 3 that a phylogenetically informed method, in this case utilizing an ancestral reconstruction, is the most rigorous approach to assessing the association between *idsA2* mutations and MICs and we thank them again for their comments.

The relevant methods section, starting at line 546, has been edited to better describe this approach. It is also included below for convenience.

“**Variant association with MIC in CRyPTIC dataset**

To assess the association of *idsA2* variants with MIC changes across the 13 drugs from the CRyPTIC dataset, we sought to determine the difference in MIC between strains resulting from *idsA2* mutational events and their nearest neighbors. First, we performed filtering on the MIC data. Only MIC values defined as high quality for the drug being analyzed were used. Due to differences in plate set-up, the extreme values were adjusted to reflect only the values available on both plates [50]. For example, UKMYC5 has an ethambutol range of 0.06-8 mg/L with minimum MIC values labeled as <=0.06 mg/L and maximum >8 mg/L. UKMYC6 has an ethambutol range of 0.25-32 mg/L with minimum MIC values labeled as <=0.25 mg/L and maximum >32 mg/L. For our analysis, all values less than or equal to 0.25 (<=0.06, 0.12, <=0.25, 0.25) from both plates were labeled as 0.25 while all values greater than or equal to 8 (8, >8, 16, 32, >32) were labeled as 8.

Next, we calculated the difference in MIC for each strain and node relative to their respective near neighbors on the phylogenetic tree. Variant calling, tree building, annotation, and variant settings are described above. The CRyPTIC tree was pruned using *dendropy* to include only strains in the lineage of interest with high quality MIC data for the drug of interest [64]. Across the whole tree, we calculated the difference in MIC between each target and sibling set, otherwise referred to as the nearest neighbor(s). By calculating a value for the difference in MIC for each strain and node, we could then use this dataset to both identify nodes with *idsA2* mutational events and permute randomly across the tree.

We identified the nodes with *idsA2* mutational events using the variant calling per our standard settings coupled with the ancestral reconstruction. Each point plotted in Figure 2 is data from a single *idsA2* mutational event. We averaged the differences in MIC across *idsA2* events to acquire the associated change in MIC for *idsA2* mutational events, visualized by the blue line in Figure 2. There are three instances in which the nearest neighbor strains to an *idsA2* event node have an *idsA2* mutant strain, however, we chose to retain these events in the dataset for consistency with the permutation test, in which we averaged the calculated differences across the same number of random nodes as *idsA2* events in that tree, 10,000 times. Significance was determined by how many times the test returned an average difference greater than the *idsA2* events. Multiple testing was corrected by two-stage linear step-up procedure of Benjamini, Krieger and Yekutieli in Graphpad Prism 10 with a 1% false discovery rate.”

2. The finding of a decrease in rifampin IC90 in idsA2 mutants is interesting, and discordant with the prediction from the phylogenetic nearest-neighbor comparisons, although as the authors point out, concordant with other work in the field. Do the authors have any hypotheses why this may occur? In particular, a hypothesis for why rifampin MIC would decrease in a idsA2 mutant background specifically, especially given the new mechanistic knowledge generated in this study? Could be worth a mention in the discussion.

We appreciate the opportunity to discuss this point. We discuss the first part, the reason for the discordance between the rifampicin phenotype in the CRyPTIC dataset and our isogenic strain assays in question 3 from Reviewer 1.

We also investigated the mechanistic reason behind the increase in rifampicin sensitivity in *idsA2* mutant strains. The Rock Lab previously published a chemical genetic screen in which they studied bacterial fitness of a panel of gene knockdowns in the presence of several drugs (Li, *et al.* 2022). In this work, they found that knockdown of multiple genes led to increased sensitivity to bedaquiline, rifampicin, and vancomycin and that these knockdown strains also have increased permeability to ethidium bromide and, more reliably, a fluorescent vancomycin conjugate. In this dataset, *idsA2* knockdown strains are also more sensitive to bedaquiline, rifampicin, and vancomycin. This finding is concordant with our data in which *idsA2* mutant strains are more sensitive to rifampicin and bedaquiline. Therefore, we tested whether our mutant strains have increased permeability. To do so, we incubated our strains in calcein AM which is cell permeable. Once inside the cell, the molecule is cleaved into fluorescent calcein which cannot escape the cell. We found that *idsA2* S115X is more permeable to calcein AM, while *idsA2* L204R is not. Further work is required to demonstrate whether the rifampicin and bedaquiline phenotypes we observe are due to an increase in permeability.

We have added this data to the manuscript at S13 Fig, also seen above. We discuss this finding in the Results starting at line 255:

“The mechanism behind this collateral sensitivity is unclear. In a CRISPRi screen, it was observed that both bedaquiline and rifampicin sensitivity increase in strains with increased permeability [21]. We tested the hypothesis that *idsA2* mutants also have increased permeability by measuring calcein accumulation in the bacterial cell. However, our findings were inconsistent between the *idsA2* mutant strains (S13 Fig). Further work is required to determine the mechanisms of these sensitivity phenotypes.”

In the Discussion starting at line 401:

“We note that the mechanistic basis of the changes in rifampicin and bedaquiline susceptibility remain unclear; we considered changes in cell wall permeability which has been previously associated with a similar set of drug phenotypes [21], but did not find consistent evidence of this in a calcein accumulation assay.”

And we describe the calein accumulation assay in detail in the Methods starting at line 775.

3. For the ordering of mutation analysis, this approach seems reasonable. However, as I understand the approach, the authors rely on maximum parsimony-based ancestral state reconstruction of the idsA2 mutational state to identify nodes defining the idsA2 mutational event (from the previous work Culviner et al., 2025: I don’t see a note if ACCTRAN or DELTRAN or DOWNPASS parsimony optimization was used, which theoretically could affect this analysis). I think it would be more consistent to use the parsimony ancestral state reconstructions for embB mutations from the same analysis. Although I don’t think it’s critical, it should also be noted this analysis might be affected by phylogenetic uncertainty, which is not taken into account.

We agree with Reviewer 3 that use of maximum parsimony-based ancestral reconstruction with DOWNPASS parsimony optimization, and the resulting phylogenetic uncertainty, can impact our results. It is for this reason that we do not use the ancestral state reconstruction to determine *embB* mutational events. Common variants, such as those which cause drug resistance in *embB*, are more likely to have multiple parsimonious solutions. Therefore, we performed a local parsimony analysis to determine the position of *embB* mutational events relative to *idsA2* mutations to avoid this uncertainty. We do not have the same concern about phylogenetic uncertainty for *idsA2* mutational event calls, because in this case there are relatively sparce mutational events with a wider variety of variant types occurring. We clarified this choice in the Methods starting at line 819:

**“Ordering analysis**

To determine the phylogenetic order of *idsA2* and *embB* mutations, we utilize the ancestral reconstruction of *idsA2* mutational events meeting the indicated variant criteria and occurring in Lineage 4 of the 55K dataset. However, *embB* repeatedly acquires the same variants making the ancestral reconstruction of *embB* events more likely to have multiple parsimonious solutions. To avoid this, we instead perform a local parsimony analysis for *embB* around *idsA2* mutational events. To do so, at each *idsA2* mutation node, we asked which of the strains descending from this event had *embB* variants…”

4. For the analysis of concomitant embB mutations in the background of idsA2 mutations (Lines 289-295), recommend reporting in text that there are no off-target (or even on-target) mutations in addition to the Q497R mutations found (I found it in Table S4)

We thank Reviewer 3 for identifying this in our supplementary information. As suggested, we now state on line 327 and 844:

“There were no additional mutations shared between strains (S4 Table).”

5. Line 460: should define acronym HGVS on first use, but defer to editor if this is common enough to not be required

We thank Reviewer 3 for pointing this out. This acronym was removed for clarity.
