# Supplementary material for: Encoded metabolic remodeling amplifies drug resistance in Mycobacterium tuberculosis
Source: PLoS Pathog. 2026 Aug 14;22(8):e1014237. doi: 10.1371/journal.ppat.1014237 (PMC13497261; doi:10.1371/journal.ppat.1014237)
Supplement: Peer review history

# Peer review: Decision Letter 0

**DOI:** 10.1371/journal.ppat.1014237.r001

**Authors:** Marcel A. Behr, Debra E Bessen

17 Jun 2026

PPATHOGENS-D-26-01095

Encoded metabolic remodeling amplifies drug resistance in *Mycobacterium tuberculosis*

PLOS Pathogens

Dear Dr. Fortune,

Thank you for submitting your manuscript to PLOS Pathogens. After careful consideration, we feel that it has merit but does not fully meet PLOS Pathogens's publication criteria as it currently stands. Therefore, we invite you to submit a revised version of the manuscript that addresses the points raised during the review process.

Please submit your revised manuscript by Jul 17 2026 11:59PM. If you will need more time than this to complete your revisions, please reply to this message or contact the journal office at plospathogens@plos.org. When you're ready to submit your revision, log on to https://www.editorialmanager.com/ppathogens/ and select the 'Submissions Needing Revision' folder to locate your manuscript file.

Please include the following items when submitting your revised manuscript:

* A letter that responds to each point raised by the editor and reviewer(s). You should upload this letter as a separate file labeled 'Response to Reviewers'. This file does not need to include responses to any formatting updates and technical items listed in the 'Journal Requirements' section below.

* A marked-up copy of your manuscript that highlights changes made to the original version. You should upload this as a separate file labeled 'Revised Manuscript with Track Changes'.

* An unmarked version of your revised paper without tracked changes. You should upload this as a separate file labeled 'Manuscript'.

If you would like to make changes to your financial disclosure, competing interests statement, or data availability statement, please make these updates within the submission form at the time of resubmission. Guidelines for resubmitting your figure files are available below the reviewer comments at the end of this letter.

As the corresponding author, your ORCID iD is verified in the submission system and will appear in the published article. PLOS supports the use of ORCID, and we encourage all coauthors to register for an ORCID iD and use it as well. Please encourage your coauthors to verify their ORCID iD within the submission system before final acceptance, as unverified ORCID iDs will not appear in the published article. Only the individual author can complete the verification step; PLOS staff cannot verify ORCID iDs on behalf of authors.

We look forward to receiving your revised manuscript.

Kind regards,

Marcel A. Behr

Academic Editor

PLOS Pathogens

Debra Bessen

Section Editor

PLOS Pathogens

Sumita Bhaduri-McIntosh

Editor-in-Chief

PLOS Pathogens

orcid.org/0000-0003-2946-9497

Michael Malim

Editor-in-Chief

PLOS Pathogens

orcid.org/0000-0002-7699-2064

**Additional Editor Comments (if provided):**

**Journal Requirements:**

If the reviewer comments include a recommendation to cite specific previously published works, please review and evaluate these publications to determine whether they are relevant and should be cited. There is no requirement to cite these works unless the editor has indicated otherwise.

1) Please send a completed 'Competing Interests' statement, including any COIs declared by your co-authors. If you have no competing interests to declare, please state "The authors have declared that no competing interests exist". Otherwise please declare all competing interests beginning with the statement "I have read the journal's policy and the authors of this manuscript have the following competing interests:"

2) Thank you for stating "All data are available in main text or supplementary materials. Sequencing data are deposited in the Short Read Archive database (Bioproject: PRJNA1356913). Phylogenetic trees, ancestral reconstruction, and variant calling were deposited to Mendeley (55K dataset DOI: 10.17632/cy4dy352kj.1, CRyPTIC dataset DOI: 10.17632/cy4dy352kj.1). Code: "https://github.com/abigailmfrey/IdsA2_Paper_Code." However, we were unable to access the sequence data using the information provided. Please review and update the Data Availability Statement to ensure that the underlying data are publicly accessible and that all accession numbers, links, or repository details are correct and functional.

**Reviewers' Comments:**

Reviewer's Responses to Questions

**Part I - Summary**

Please use this section to discuss strengths/weaknesses of study, novelty/significance, general execution and scholarship.

Reviewer #1: In this study Frey et al. follow up on their earlier identification of idsA2 as a frequent target of within-host selection and use a combination of genomic, phenotypic, and biochemical analyses to characterize idsA2 loss of function mutations (as identified from human clinical datasets) as contributors to ethambutol resistance. The major strengths of this study include not only the combination of bioinformatic and experimental analyses that lend strong support to their conclusions, but also the extension into potential clinical relevance via assessing the potential of idsA2 variants to improve ethambutol resistance screening. Overall, these qualities make the study of high interest and value to the PLOS Pathogens audience. Detailed comments are largely minor and pertain to questions of rigor, sometimes related to arguably confusing turns of phrase.

Reviewer #2: In brief, Frey et al. report a novel association/ enrichment of mutations in the idsA2 gene for Lineage 4 Mycobacterium tuberculosis clinical strains with the potential to reduce the sensitivity of these strains towards certain anti-TB antibiotics, particularly ethambutol (EMB). In turn, targeted lipid analysis led the authors to demonstrate an effect on isoprene precursor metabolism that may help to explain this reduction in EMB sensitivity. To my mind, highlighting the potential for lineage/ sub-lineage metabolic differences that can impact treatment outcomes is, perhaps, the most important message contained within this manuscript. By extension, this well-executed study may help to point us towards the development and testing of “personalized” lineage-specific regimens in the future, rather than continuing with the one treatment fits-all approach that has been the cornerstone of TB treatment for the past decades.

Reviewer #3: In this original research article, Frey et al. investigate the significance of idsA2 mutations on antimicrobial resistance in the M. tuberculosis complex. IdsA2 had previously been identified to be under directional selection in the authors' previous work to develop a per-gene and per-lineage pN/pS ratio database. The authors first present evidence that this gene is under diversifying selection for loss of function. They then used the CRyPTIC MIC dataset and compared phylogenetic nearest neighbors to test for association between idsA2 mutations and MIC differences, finding associations with rifampin, isoniazid and ethambutol. They then sought to understand two of these mutations using recombineering to place them in an H37Rv background. These idsA2 mutants had a slight growth defect, but higher IC90s to isoniazid and ethambutol and a lower IC90 to rifampin. They then characterize the mutations and their effects on ethambutol binding. Finally, they show that phylogenetically these idsA2 mutations tended to occur after embB mutations, and lead to a combinatorial increase in ethambutol MIC. The consideration of mutations in both genes is then shown to increase the specificity of genotypic ethambutol susceptibility testing.

Overall, I believe this is a well designed, executed and communicated manuscript. I believe the methods are generally appropriate and the results support the authors’ conclusions. I do have some comments and suggestions to further improve the manuscript.

**********

**Part II – Major Issues: Key Experiments Required for Acceptance**

Please use this section to detail the key new experiments or modifications of existing experiments that should be absolutely required to validate study conclusions.

Generally, there should be no more than 3 such required experiments or major modifications for a "Major Revision" recommendation. If more than 3 experiments are necessary to validate the study conclusions, then you are encouraged to recommend "Reject".

Reviewer #1: None requested.

Reviewer #2: (No Response)

Reviewer #3: The GitHub repository is not currently active to review the details of the nearest-neighbor approach described on lines 477-498. I did not quite understand how exactly this analysis is performed. If the analysis is comparing the MICs of a subtree subtended by a node defined as having an ancestral state with an idsA2 mutation to the nearest neighbor – is there any check to ensure that the nearest neighbor subtree (however defined) does not also contain an idsA2 mutation somewhere within it? Apologies if I have misunderstood the approach, perhaps it can be clarified in the methods text. Could a phylogenetic comparative method also be useful here to address the question?

**********

**Part III – Minor Issues: Editorial and Data Presentation Modifications**

Please use this section for editorial suggestions as well as relatively minor modifications of existing data that would enhance clarity.

Reviewer #1: Line 121: “SIFT-predicted deleterious missense mutation” Since SIFT is arguably not a widely familiar tool (vs. e.g., something like BLAST), suggest removing, briefly describing, or referring to methods for clarity.

Line 131: which --> that. Please check overall for correct use of these relative pronouns.

Line 136, Fig. 2 and Line 167, Fig. 3. There appears to be a discrepancy in the RIF sensitivity of idsA2 mutational events as determined from the CRyPTIC dataset (more resistant; Fig. 2) vs. from individual mutants (more sensitive; Fig. 3A, D). Could the authors please comment?

Related, what assay was used to the determine MIC in the CRyPTIC dataset? Could any differences explain the RIF discrepancy? This study used Alamar Blue, which reports on respiration but may not report reliably on viability depending on metabolic state. Given that idsA2 mutations affect energy/metabolic status, did the authors determine MIC, especially for EMB, by an independent assay?

Line 196: “concentrations in metabolite [] from wildtype and…” Word appears to be missing at brackets? Metabolite extracts? Related also to Line 214, which refers to analysis of “whole-cell lysates”. Methods report lysis/extraction in acetonitrile:MeOH:water. Suggest adding/modifying wording for clarity and consistency throughout.

Line 258: Please clarify briefly for non-expert readers why DPP is a combined measure of DPA and DPR and how later conclusions based on inference of DPA levels is valid.

Lines 290-292: The methodology of generating the double mutants is somewhat confusingly worded, as idsA2 mutations were generated by recombination, but the embB mutation by selection and, importantly, sequencing. Please modify for clarity.

Lines 293-296: Would resistance-conferring mutations be expected to increase MICs by a fixed concentration amount, or by fold change? On both backgrounds, addition of idsA2 mutations increase MIC by 2-fold. Please comment and possibly modify the “however” in line 295 accordingly.

Related, line 425: “Most notably, idsA2 mutations have a multiplicative effect with embR variants on [EMB] resistance.” (and similar in abstract, line 35) As noted above, they have a multiplicative effect on WT background also. Please modify/clarify accordingly.

Line 336: “presence of… an idsA2 mutation…” Fig. 7A shows analysis for embB mutant, but not idsA2 mutant. Are the data for this conclusion (lower false positive rate with inclusion of idsA2 mutant alone) shown anywhere? Please clarify.

Line 353: “This pathway remodeling also enables its most clinically relevant effect” The most relevant connection to resistance is the model that increases in DPA compete for ethambutol binding to embB, but this is not directly demonstrated. Suggest modulating wording for correlation vs. causation.

On that note, lines 33-34: “increased production of [DPP], which can compete with ethambutol for binding to arabinosyltransferases” vs. line 262: “it was proposed that… increasing the quantity of DPA allows arabinose to outcompete ethambutol for binding” Is there in fact experimental evidence from any previous study for this competition? If not, suggest modifying abstract accordingly. Also please note and consider resolving the discrepancy/confusion regarding referring to DPP as the actual measure vs. DPA as the specific relevant metabolite (see related comment above).

Line 423: “through the pleiotropic effects of metabolite remodeling” We feel that this wording is reasonable given the data, but confusing in this manuscript due to the previous emphasis in abstract and main text on the inferred contribution of elevated DPA to ethambutol inhibition of the EmbB target. A minor point, but possibly combining these would be most clear—that is, there are pleiotropic effects, of which elevated DPP/DPA provides the most direct connection to EMB mechanism of action. Please consider modifying for consistency and accuracy, with advance thanks for helping especially non-expert readers achieve the clearest possible sense of the conclusions.

Reviewer #2: Suggestions for improving the manuscript:

The abstract should make it clear that the impact of idsA2 mutation(s) on EMB resistance appears restricted to strains within lineage 4. Similarly, the increase in MIC (or more correctly IC90) is relatively small and in the order of 2-fold. Personally, I think the abstract oversells the case for idsA2, particularly with statements such as “Together, this work defines idsA2 as an ethambutol resistance gene”. By itself, idsA2 does not appear to confer EMB resistance. Please temper this statement to more accurately reflect the reality.

Are the frequencies of EMB-R strains comparable between the 4 Mtb lineages that make up the 55K database? Please comment in the text on this.

Regarding the CRyPTIC dataset, 19 lineage 4 strains seem to have a predicted deleterious idsA2 mutation (Figure S4). How many of these also have a canonical mutation in embB? Is it possible to indicate which strains have an embB mutation on the relevant panel of figure S4?

Lines 133-134: “We calculated the average difference in MICs between Lineage 4 strains with idsA2 mutations ….”. What is the result if this same calculation is performed for lineage 1-3 strains?

Unfortunately, aside from the standard fall-back H37Rv lab strain, the authors do not seem to have carried out the in vitro idsA2 recombineering with a non-lineage 4 strain to examine its effect on isoprene metabolism and/ or the EMB IC90. A lack of an effect on isoprene metabolism (with a corresponding lack of EMB MIC shift) would have gone a long way towards supporting the author’s suggestion that the effect is truly lineage specific.

Lines 295-297: In my opinion, this material is written in a somewhat misleading/deceptive manner. The relative increase in IC90 due to the idsA2 mutations is still only 2-fold, regardless of whether they co-occur with a canonical embB mutation(s) responsible for conferring EMB-R. In fact, what has caused the 8-fold shift in IC90 is the embB mutation, ie. 0.5 ug/ml to 4ug ug/ml in the absence of the idsA2 SNP; 1ug/ml to 8ug/ml in the presence of the idsA2 SNP.

Reviewer #3: - The finding of an a decrease in rifampin IC90 in idsA2 mutants is interesting, and discordant with the prediction from the phylogenetic nearest-neighbor comparisons, although as the authors point out, concordant with other work in the field. Do the authors have any hypotheses why this may occur? In particular, a hypothesis for why rifampin MIC would decrease in a idsA2 mutant background specifically, especially given the new mechanistic knowledge generated in this study? Could be worth a mention in the discussion.

- For the ordering of mutation analysis, this approach seems reasonable. However, as I understand the approach, the authors rely on maximum parsimony-based ancestral state reconstruction of the idsA2 mutational state to identify nodes defining the idsA2 mutational event (from the previous work Culviner et al., 2025: I don’t see a note if ACCTRAN or DELTRAN or DOWNPASS parsimony optimization was used, which theoretically could affect this analysis). I think it would be more consistent to use the parsimony ancestral state reconstructions for embB mutations from the same analysis. Although I don’t think it’s critical, it should also be noted this analysis might be affected by phylogenetic uncertainty, which is not taken into account.

- For the analysis of concomitant embB mutations in the background of idsA2 mutations (Lines 289-295), recommend reporting in text that there are no off-target (or even on-target) mutations in addition to the Q497R mutations found (I found it in Table S4)

- Line 460: should define acronym HGVS on first use, but defer to editor if this is common enough to not be required

**********

PLOS authors have the option to publish the peer review history of their article (what does this mean?). If published, this will include your full peer review and any attached files.

If you choose “no”, your identity will remain anonymous but your review may still be made public.

**Do you want your identity to be public for this peer review?**  For information about this choice, including consent withdrawal, please see our Privacy Policy.

Reviewer #1: No

Reviewer #2: No

Reviewer #3: No

[NOTE: If reviewer comments were submitted as an attachment file, they will be attached to this email and accessible via the submission site. Please log into your account, locate the manuscript record, and check for the action link "View Attachments". If this link does not appear, there are no attachment files.]

**Figure resubmission:**

While revising your submission, we strongly recommend that you use PLOS’s NAAS tool (https://ngplosjournals.pagemajik.ai/artanalysis) to test your figure files. NAAS can convert your figure files to the TIFF file type and meet basic requirements (such as print size, resolution), or provide you with a report on issues that do not meet our requirements and that NAAS cannot fix.

After uploading your figures to PLOS’s NAAS tool - https://ngplosjournals.pagemajik.ai/artanalysis, NAAS will process the files provided and display the results in the "Uploaded Files" section of the page as the processing is complete. If the uploaded figures meet our requirements (or NAAS is able to fix the files to meet our requirements), the figure will be marked as "fixed" above. If NAAS is unable to fix the files, a red "failed" label will appear above. When NAAS has confirmed that the figure files meet our requirements, please download the file via the download option, and include these NAAS processed figure files when submitting your revised manuscript.

**Reproducibility:**

To enhance the reproducibility of your results, we recommend that authors of applicable studies deposit laboratory protocols in protocols.io, where a protocol can be assigned its own identifier (DOI) such that it can be cited independently in the future. Additionally, PLOS ONE offers an option to publish peer-reviewed clinical study protocols. Read more information on sharing protocols at https://plos.org/protocols?utm_medium=editorial-email&utm_source=authorletters&utm_campaign=protocols

# Author comment: Author response to Decision Letter 1

**DOI:** 10.1371/journal.ppat.1014237.r002

16 Jul 2026

Attachment Submitted filename: Response to Reviewers.docx

# Peer review: Decision Letter 1

**DOI:** 10.1371/journal.ppat.1014237.r003

**Authors:** Marcel A. Behr, Debra E Bessen

4 Aug 2026

Dear Dr. Fortune,

We are pleased to inform you that your manuscript 'Encoded metabolic remodeling amplifies drug resistance in *Mycobacterium tuberculosis* ' has been provisionally accepted for publication in PLOS Pathogens.

Before your manuscript can be formally accepted you will need to complete some formatting changes, which you will receive in a follow up email. A member of our team will be in touch with a set of requests.

Please note that your manuscript will not be scheduled for publication until you have made the required changes, so a swift response is appreciated.

IMPORTANT: The editorial review process is now complete. PLOS will only permit corrections to spelling, formatting or significant scientific errors from this point onwards. Requests for major changes, or any which affect the scientific understanding of your work, will cause delays to the publication date of your manuscript.

Should you, your institution's press office or the journal office choose to press release your paper, you will automatically be opted out of early publication. We ask that you notify us now if you or your institution is planning to press release the article. All press must be co-ordinated with PLOS.

Thank you again for supporting Open Access publishing; we are looking forward to publishing your work in PLOS Pathogens.

Best regards,

Marcel A. Behr

Academic Editor

PLOS Pathogens

Debra Bessen

Section Editor

PLOS Pathogens

Sumita Bhaduri-McIntosh

Editor-in-Chief

PLOS Pathogens

orcid.org/0000-0003-2946-9497

Michael Malim

Editor-in-Chief

PLOS Pathogens

orcid.org/0000-0002-7699-2064

***********************************************************

Reviewer Comments (if any, and for reference):

Reviewer's Responses to Questions

**Part I - Summary**

Please use this section to discuss strengths/weaknesses of study, novelty/significance, general execution and scholarship.

Reviewer #3: In this revised original research article, Frey et al. investigate the significance of idsA2 mutations on antimicrobial resistance in M. tuberculosis complex. The main conclusions of the articles are that idsA2 is found to be under diversifying selection in lineage 4, and that mutations in this gene lead to a modest but detectable increase in MIC to ethambutol, and are found to have evolved after embB mutations leading to higher level resistance. The mechanism is investigated and is argued to be increased production of decaprenyl phosphate species.

In this revision, the authors have addressed the concerns that myself and the other reviewers have raised. Overall, I find the revised article more clear and with a more careful qualification of the conclusions.

Focusing on the issues that I had previously raised:

Nearest-neighbor approach: The expanded description provided by the authors of the approach (and the github scripts) have clarified the approach. I think this is a reasonable approach as explained, and also think the authors have improved the framing of these results around lines 149-152 (marked-up copy).

Other comments:

- The expanded discussion of decreased rifampin MIC in idsA2 mutants in response to my and the other reviewers’ comments, including the new Figure S13 and calcein accumulation assay is appreciated.

- The additional clarity concerning the local parsimony analysis given in lines 820-830 (marked up copy) is also appreciated and I think the justification of the author's approach is sound with respect to the conclusions drawn.

**********

**Part II – Major Issues: Key Experiments Required for Acceptance**

Please use this section to detail the key new experiments or modifications of existing experiments that should be absolutely required to validate study conclusions.

Generally, there should be no more than 3 such required experiments or major modifications for a "Major Revision" recommendation. If more than 3 experiments are necessary to validate the study conclusions, then you are encouraged to recommend "Reject".

Reviewer #3: see above

**********

**Part III – Minor Issues: Editorial and Data Presentation Modifications**

Please use this section for editorial suggestions as well as relatively minor modifications of existing data that would enhance clarity.

Reviewer #3: see above

**********

PLOS authors have the option to publish the peer review history of their article (what does this mean?). If published, this will include your full peer review and any attached files.

If you choose “no”, your identity will remain anonymous but your review may still be made public.

**Do you want your identity to be public for this peer review?**  For information about this choice, including consent withdrawal, please see our Privacy Policy.

Reviewer #3: No

# Editor report: Acceptance letter

**DOI:** 10.1371/journal.ppat.1014237.r004

**Authors:** Marcel A. Behr, Debra E Bessen

Dear Dr. Fortune,

We are delighted to inform you that your manuscript, "Encoded metabolic remodeling amplifies drug resistance in *Mycobacterium tuberculosis* ," has been formally accepted for publication in PLOS Pathogens.

We have now passed your article onto the PLOS Production Department who will complete the rest of the pre-publication process. All authors will receive a confirmation email upon publication.

The corresponding author will soon be receiving a typeset proof for review, to ensure errors have not been introduced during production. Please review the PDF proof of your manuscript carefully, as this is the last chance to correct any scientific or type-setting errors. Please note that major changes, or those which affect the scientific understanding of the work, will likely cause delays to the publication date of your manuscript. Note: Proofs for Front Matter articles (Pearls, Reviews, Opinions, etc...) are generated on a different schedule and may not be made available as quickly.

Soon after your final files are uploaded, the early version of your manuscript, if you opted to have an early version of your article, will be published online. The date of the early version will be your article's publication date. The final article will be published to the same URL, and all versions of the paper will be accessible to readers.

For Research Articles, you will receive an invoice from PLOS for your publication fee after your manuscript has reached the completed accept phase. If you receive an email requesting payment before acceptance or for any other service, this may be a phishing scheme. Learn how to identify phishing emails and protect your accounts at https://explore.plos.org/phishing.

Thank you again for supporting open-access publishing; we are looking forward to publishing your work in PLOS Pathogens.

Best regards,

Sumita Bhaduri-McIntosh

Editor-in-Chief

PLOS Pathogens

orcid.org/0000-0003-2946-9497

Michael Malim

Editor-in-Chief

PLOS Pathogens

orcid.org/0000-0002-7699-2064
